# Genome-Wide Transcriptome Analysis Reveals GRF Transcription Factors Involved in Methyl Jasmonate-Induced Flavonoid Biosynthesis in *Hedera helix*

**DOI:** 10.3390/plants14142094

**Published:** 2025-07-08

**Authors:** Feixiong Zheng, Zhangting Xu, Xiaoji Deng, Xiaoyuan Wang, Yiming Sun, Xiaoxia Shen, Zhenming Yu

**Affiliations:** 1 School of Pharmaceutical Sciences, Zhejiang Chinese Medical University, Hangzhou 310053, China; feixiongz@163.com (F.Z.); 15168104031@163.com (Z.X.); 13868867263@163.com (X.D.); 18268356984@163.com (X.W.); 20221013@zcmu.edu.cn (Y.S.); 2 Songyang Institute, Zhejiang Chinese Medical University, Lishui 323400, China

**Keywords:** *Hedera helix*, flavonoid, transcriptome, MeJA, growth-regulating factor

## Abstract

Flavonoids are key bioactive compounds in plants that play important defense roles against abiotic stress and are involved in plant growth and development. Methyl jasmonate (MeJA) is a significant growth regulator that promotes the accumulation of flavonoids in a variety of plants, but the effect of MeJA in *Hedera helix* remains poorly understood. In the present study, the flavonoid content was significantly increased after MeJA treatment and peaked at 6 h post-treatment. A total of 31,931 genes were identified using transcriptome, and 6484 DEGs were identified at 6 h post-treatment. Through GO and KEGG enrichment analysis, it was shown that DEGs were primarily enriched in phenylpropanoid biosynthesis pathways. Based on the putative transcription factors derived from DEGs, growth-regulating factor (GRF), a transcription factor potentially linking MeJA signaling to flavonoid accumulation and participating in plant growth and stress responses, was further identified. A total of 20 Hh-GRFd genes were identified on the whole genome level and clustered into five phylogenetic groups with conserved subfamily characteristics. Abundant MeJA-responsive cis-elements were presented in the promoter regions of *HhGRF1*-*HhGRF20*. They exhibited a tissue-specific expression variation, and *HhGRF10* was dominantly expressed in leaves of *H. helix*. Notably, *HhGRF10* exhibited MeJA-induced expression that correlated temporally with flavonoid accumulation, suggesting that *HhGRF10* might play a potential role in promoting flavonoid biosynthesis, and overexpression and knockout assay substantiated this conclusion. The finding provides the first transcriptome-wide resource for flavonoid biosynthesis in *H. helix* and identifies the candidate GRF-mediated regulator for flavonoid accumulation.

## 1. Introduction

*Hedera helix*, as a perennial climbing plant of the genus Hedera within the family Araliaceae, is valued for its diverse bioactive compounds, including triterpenoid saponin, and flavonoids, and their leaves are the primary organ harboring these abundant metabolites [1]. In many regions of Europe, extracts derived from *H. helix* have been utilized in herbal medicinal products for treating respiratory disorders, including cough and bronchitis, owing to their potent expectorant and bronchodilatory properties [2,3]. Previous studies have predominantly focused on elucidating the biosynthetic mechanisms of saponins in this species, while research on flavonoid compounds remains notably scarce. Flavonoids represent a crucial class of plant secondary metabolites that plays vital roles not only in human health but also in plant growth, development, and stress stimulus [4]. As a crucial substance within plants, flavonoids play crucial roles in abiotic stress resistance. Acting as UV filters, flavonoids protect plants from harmful ultraviolet radiation. They can also scavenge reactive oxygen species (ROS) to mitigate oxidative stress [5]. For instance, flavonoids can regulate drought and salt responses by removing ROS and promoting the activities of antioxidant enzymes, thereby improving drought and salt stress tolerance [6].

Flavonoid biosynthesis originates from the phenylpropanoid pathway, in which phenylalanine is sequentially converted by the action of PAL, C4H, and 4CL to 4-coumaroyl-CoA, a key intermediate for flavonoid synthesis [7]. Then, the 4-Coumaroyl-CoA serves as the substrate that is converted by CHS into naringenin chalcone. Subsequently, CHI catalyzes the isomerization of this intermediate to form naringenin, the core flavanone structure that serves as a metabolic branch point for multiple flavonoid biosynthetic pathways [8]. Naringenin metabolism governs flavonoid diversity. F3H hydroxylates naringenin to form dihydroflavonols, which are channeled into distinct pathways. For anthocyanin biosynthesis, DFR produces leucoanthocyanidins, subsequently oxidized by ANS into anthocyanidins and stabilized by arGST [9]. Alternatively, FLS redirects dihydroflavonols to generate flavonols (e.g., kaempferol, quercetin). Leucoanthocyanidins may also enter the proanthocyanidin pathway via the action of LAR or ANR, yielding catechins and epicatechins, precursors for condensed tannins [10].

TFs can regulate flavonoid biosynthesis by controlling the activity of the key enzymes mentioned above. For example, MYBs not only modulate flavonoid biosynthesis through the MYB–bHLH–WD protein (MBW) complex in “late” steps (e.g., anthocyanin and proanthocyanidin biosynthesis) [11] but also exhibit bHLH-independent regulatory mechanisms in “early” steps (e.g., general phenylpropanoid and flavonol biosynthesis) [12]. In *Solanum lycopersicum*, SlbHLH95 interacts with SlMYB12 to activate *SlF3H* and *SlFLS*, the key genes of flavonoid biosynthesis [13]. SbMYB3 directly activates *SbFNSII-2* to promote root-specific flavonoid biosynthesis in *Scutellaria baicalensis* [14]. In addition to MYBs, other TFs also play regulatory roles in flavonoid biosynthesis. The StWRKY41 activates F3’H to enhance cold tolerance by promoting flavonoid biosynthesis and antioxidant activity in potato [15]. In *Camellia sinensis*, CsNAC086 binds to the promoter region of *CsFLS* and activates its expression, thereby promoting flavonol accumulation [16].

MeJA, a pivotal phytohormone, regulates plant development and secondary metabolism (e.g., flavonoid biosynthesis) and orchestrates stress response under adverse conditions, promoting flavonoid accumulation as a defensive strategy. In *Dioscorea composita*, MeJA significantly promotes the accumulation of proanthocyanidins (PAs). MeJA upregulates the expression of *DcWRKY11*, which directly binds to the promoter of *TT2*, thereby activating its expression and subsequently regulating PAs accumulation [17]. MeJA treatment triggers cytokinin production and activates JA signaling to induce starch and sucrose metabolism, thereby significantly enhancing total flavonoid glycoside accumulation in *Ficus pandurata* [18].

Growth-regulating factor (GRF) is a pivotal class of transcriptional regulators of plant growth and development (e.g., plant cell proliferation and expansion, leaf senescence) [19]. GRFs characteristically possess two functionally distinct yet evolutionarily conserved N-terminal domains: the QLQ domain (Glutamine-Leucine-Glutamine motif), which mediates protein–protein interactions within transcriptional regulatory complexes, and WRC domain (Tryptophan-Arginine-Cysteine motif), which confers DNA-binding specificity to downstream target genes [20]. Since the first member of GRF gene was reported in rice (*Oryza sativa*, *OsGRF1*) [21], the GRF gene family has been systematically identified in different terrestrial plants, including model plants (*Arabidopsis thaliana* [22], *Nicotiana tabacum* [23]), important crops (*Zea mays* [24]), economically significant fruit (*Citrus sinensis* [25]), and major agricultural commodities (*Glycine max* [26], *S. lycopersicum* [27]).

With the evolution of GRF functions, its roles in hormone signal transduction and secondary metabolite biosynthesis are demonstrated. GRFs are induced by exogenous hormones, which are indispensable for plant growth and the production of specialized metabolites. In *Panax ginseng*, the GRF gene family, particularly *PgGRF7*, exhibits differential responsiveness to exogenous hormones (e.g., 6-BA, ABA, GA3, and IAA) [28]. In *Solanum lycopersicum*, PmGRF7 modulates leaf and pollen development through ABA and GA hormone signaling, concurrently enhancing chlorophyll accumulation [29]. Similarly, in *Oryza sativa*, *OsGRF7* responds to exogenous IAA and GA3 hormone induction, leading to a thickening of the stem cell wall [30]. In *Solanum tuberosum*, StGRFs show upregulated expression under ABA, GA3, IAA, and BAP treatments, thereby regulating the dormancy-to-sprouting transition of tubers [31]. Even though GRFs are firstly characterized as growth regulators, emerging evidence suggests they are associated with flavonoid synthesis. For instance, the silencing of *PeGRF6* in *Phalaenopsis equestris* through VIGS led to a conspicuous reddening of newly developed leaves, accompanied by significantly elevated (threefold) anthocyanin levels compared to control plants [32]. IbGRF9 promotes anthocyanin accumulation by directly binding to the promoters of anthocyanin biosynthetic genes (*IbCHS*, *IbF3H*, *IbANS*, and *IbUFGT*) and activating their transcription in sweet potato [33].

Currently, flavonoid biosynthesis is well-characterized in medicinal herbs; however, its regulation in perennial evergreens like *H. helix* remains unclear. This gap is critical to *H. helix*, which exhibits exceptional stress resilience and expanded flavonoid accumulation, yet studies on the regulatory genes and hormonal inducers of flavonoid biosynthesis remain limited. Therefore, the systematic identification and characterization of pivotal transcription factors governing flavonoid biosynthesis will contribute more to the improvement of abiotic stress resilience and medicinal compound accumulation in *H. helix*. To investigate MeJA-induced flavonoid biosynthesis in *H. helix*, the leaves of *H. helix* were treated with MeJA for flavonoid detection and transcriptomic analysis. Subsequently, the GRF gene family was characterized, and *HhGRF10* was functionally verified. This study establishes a foundational framework for elucidating the molecular mechanisms underlying flavonoid biosynthesis in *H. helix*.

## 2. Results

### 2.1. Accumulation of Total Flavonoid in H. helix Leaves Following MeJA Induction

**Figure 1:**
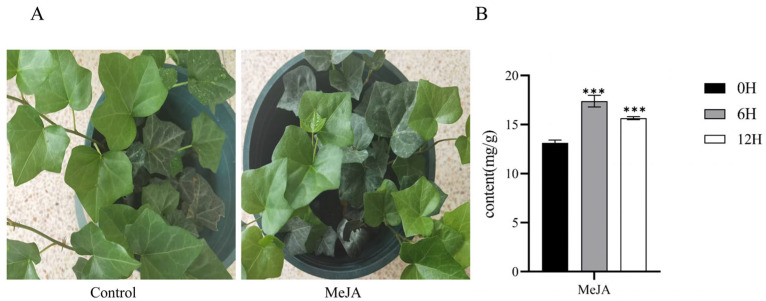
(**A**) Phenotype of control and methyl jasmonate (MeJA)-treated *H*. *helix* plants. (**B**) Flavonoid accumulation in MeJA-treated *H. helix* at 0, 6, and 12 h post-treatment. Significant differences: *** (*p* < 0.001).

The total flavonoid content in *H. helix* leaves (Figure 1A) at different times after MeJA treatment was quantified. Flavonoid accumulation peaked at 6 h post-treatment, followed by a gradual decline, though levels remained markedly higher than in the control group (0 h) (Figure 1B). These results demonstrate that MeJA effectively promotes flavonoid and accumulation in *H. helix*.

### 2.2. Transcriptome Sequencing Assembly and DEGs Identification

cDNA libraries were first constructed using total RNA from nine groups, followed by Illumina sequencing and eukaryotic reference-based transcriptome (RNA-seq) analysis. A total of 63.23 Gb of Clean Data was obtained, with each sample yielding ≥ 6.88 Gb (Table S1). The Q30 base percentage exceeded 92.85%, demonstrating the high-throughput and high-quality nature of the RNA-seq data. After adapter trimming and low-quality sequence removal, the Clean Reads from each sample were aligned to the *H. helix* genome, achieving mapping efficiencies in the range of 87.38–90.35%. Based on the alignment results, alternative splicing prediction, gene structure refinement, and new gene discovery were performed. In total, 31,931 genes were identified, including 11,376 new genes, of which 5986 were functionally annotated.

**Figure 2:**
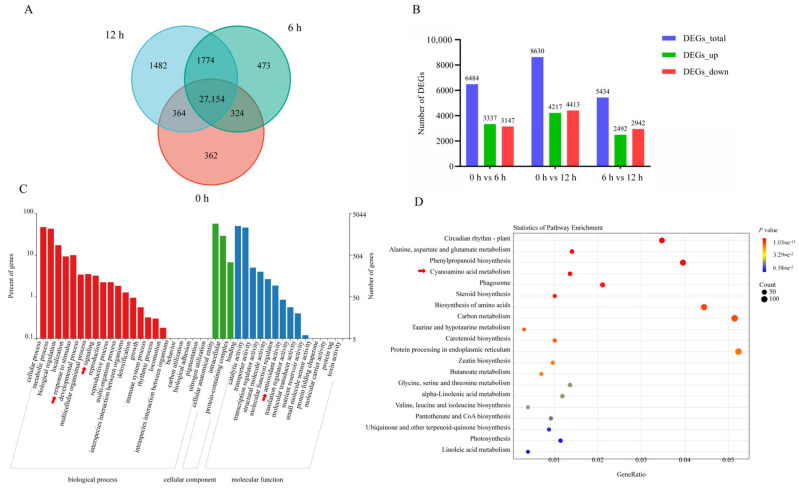
Transcriptomic profiling of MeJA-treated *H*. *helix*. (**A**) Number of DEGs at each treatment time. (**B**) DEGs between control and MeJA-treated samples. (**C**) Gene Ontology (GO) term enrichment of DEGs. (**D**) KEGG pathway enrichment analysis. The red arrows represent enrichment related to flavonoid biosynthesis.

Principal component analysis (PCA) revealed significant shifts in gene expression following MeJA treatment, with clear separation between the control (0 h) and treated groups (6 h and 12 h) (Figure S1). The three groups exhibited distinct transcriptional profiles, while biological replicates within each group were tightly clustered, demonstrating high reproducibility. The first two principal components (PC1 and PC2) accounted for 39.99% and 20.66% of the total variance, respectively. In the untreated (0 h) MeJA samples, 28,202 genes were identified, while 29,723 and 30,772 genes were detected at 6 h and 12 h post-treatment, respectively (Figure 2A). Using a threshold of |Fold Change| ≥ 1.2 and FDR < 0.01, differentially expressed genes (DEGs) were screened in all three groups (Figure 2B). Compared to the control group, 6484 DEGs were identified at 6 h, including 3337 upregulated and 3147 downregulated genes. At 12 h, 8630 DEGs were detected, comprising 4217 upregulated and 4413 downregulated genes, which demonstrates an escalating impact of MeJA treatment on *H. helix.*

### 2.3. GO Enrichment and KEGG Pathway Analysis of the DEGs

The GO enrichment analysis of DEGs revealed not only the various functional categorization into biological processes, cellular components, and molecular functions, but, more importantly, it highlighted specific metabolic process induced by MeJA treatment (Figure 2C). The significant enrichment of flavonoid metabolic (GO:0009812) and biosynthetic processes (GO:0009813) within biological processes directly correlates with our experimental focus, suggesting an activation of flavonoid accumulation. Interestingly, the enrichment of lipid metabolic processes (GO:0006629) may reflect the correlation of secondary metabolite with membrane biogenesis, as flavonoids are known to accumulate in cellular compartments (Figure S2). The molecular function showed antioxidant activity, consistent with the role of flavonoids as ROS scavengers, while the enrichment of signaling implies a complex regulatory network under MeJA-induced flavonoid accumulation in *H. helix*.

Further KEGG pathway enrichment analysis of the DEGs indicated their involvement in 20 distinct pathways (Figure 2D). Among these, fundamental metabolic pathways which were essential for plant growth and development were significantly enriched (*p* < 0.05). Notably, the phenylpropanoid biosynthesis pathway (ko00940) was significantly enriched (*p* < 0.05) in MeJA-treated *H. helix*, indicating its pivotal role in mediating the plant’s response to MeJA hormonal signaling. As a central hub of plants’ secondary metabolism, this pathway not only provides essential precursors (e.g., p-coumaroyl-CoA) for flavonoid biosynthesis but also contributes to the production of diverse phenolic compounds, including lignin and anthocyanins. The specific enrichment of this pathway, rather than a general upregulation of all secondary metabolic routes, indicates a precise MeJA regulation of flavonoid metabolism in *H. helix*, potentially explaining the observed flavonoid accumulation patterns. These results demonstrate that MeJA preferentially activates the phenylpropanoid–flavonoid biosynthetic network in *H. helix*, coordinating both hormone responses and the targeted production of flavonoid.

### 2.4. Identification of GRF Gene Family in H. helix

In addition, 13 TFs were predicted among the DEGs (Figure S3), namely bHLH, WRKY, NAC, bZIP, C2H2, MYB, GRF, GRAS, HD-ZIP, Dof, TCP, C3H, and AUX/IAA. Moreover, the number of DEG members from some extensive TF families represents only a small fraction of their total family size (e.g., only 17 MYBs were identified as DEGs, despite the MYB family likely comprising hundreds of members). In striking contrast, the relatively small GRF family (with only 20 members) showed both a high ranking in significance and a substantial proportion of DEGs. Therefore, an in-depth investigation of GRF was conducted to elucidate their regulatory mechanisms in flavonoid accumulation.

A total of 20 putative HhGRF members were discovered in the *H. helix* whole genome, characterized by the conserved QLQ and WRC domains. They were defined as HhGRF1-HhGRF20.

### 2.5. Phylogenetic Analysis of HhGRFs and Other Plant GRF Proteins

**Figure 3:**
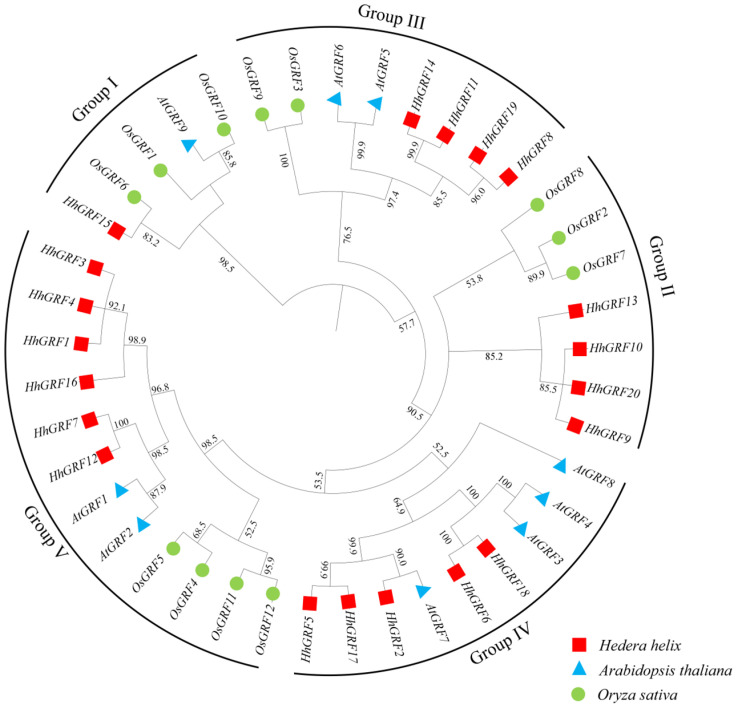
Phylogenetic tree of GRF proteins in *Arabidopsis thaliana*, *Oryza sativa*, *and Hedera helix*.

The phylogenetic analysis of GRF homologs from *H. helix*, *A. thaliana*, and *O. sativa* not only reveals evolutionary relationships but also provides insights into the potential functional diversification of HhGRFs. The clustering of HhGRFs into five different groups (Group I–V) suggests lineage-specific evolution, particularly evident in Group II (exclusive to *H. helix* and *O. sativa*) and Group IV (conserved in eudicots but absent in *O. sativa*) (Figure 3). The absence of *A. thaliana* homologs in Group II implies that these HhGRFs may have arisen from gene duplication events followed by functional specialization in *H. helix*, possibly linked to its unique environment adaptations. The expansion of Group V, the largest group in *H. helix*, could reflect selective pressure for enhanced regulatory capacity, potentially including flavonoid biosynthesis. The closer phylogenetic relationship between *H. helix* and *A. thaliana* (compared to *O. sativa*) aligns with their dicot ancestry, yet the specific absence suggests functional divergence. For instance, HhGRFs in Group II may have evolved novel roles in *H. helix*, possibly contributing to its expanded flavonoid regulatory function. Similarly, the eudicot-specific conservation of Group IV suggests these genes may promote flavonoid accumulation.

### 2.6. Chromosome Localization and Collinearity Analysis of HhGRFs Members

**Figure 4:**
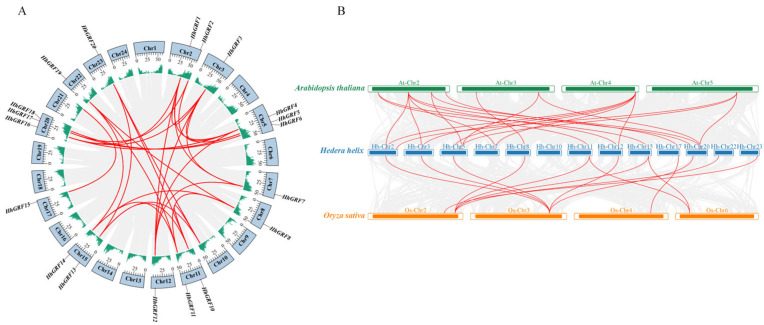
Gene replication events (**A**) of HhGRF in *Hedera helix* and (**B**) Collinearity analysis of GRF genes in *Hedera helix*, *Arabidopsis thaliana*, and *Oryza sativa.* The red line indicates collinearity between gene pairs.

Chromosome-level genome analysis of *H. helix* revealed an uneven distribution of all 20 HhGRF genes (*HhGRF1*-*HhGRF20*) across 13 chromosomes (Figure S4), with the highest density observed on Chr5 and Chr20 (3 genes). Intraspecies synteny analysis identified 29 segmental duplication events (Figure 4A), among which *HhGRF3* exhibited the most extensive collinearity relationships (five gene pairs), suggesting its preferential retention during recent gene duplication events.

Meanwhile, comparative synteny analysis among *H. helix*, *A. thaliana*, and *O. sativa* uncovered the divergent evolutionary relationship of GRF genes. The interspecies collinearity mapping revealed significantly more conserved homologous gene pairs between *H. helix* and *A. thaliana* (21 pairs) than between *H. helix* and *O. sativa* (13 pairs) (Figure 4B), demonstrating a stronger genomic conservation of GRF genes within dicots. These results provide evidence for the stronger evolutionary conservation of HhGRF genes in dicots compared to monocot lineages.

### 2.7. Conserved Motifs and Gene Structure Analysis of HhGRFs Members

**Figure 5:**
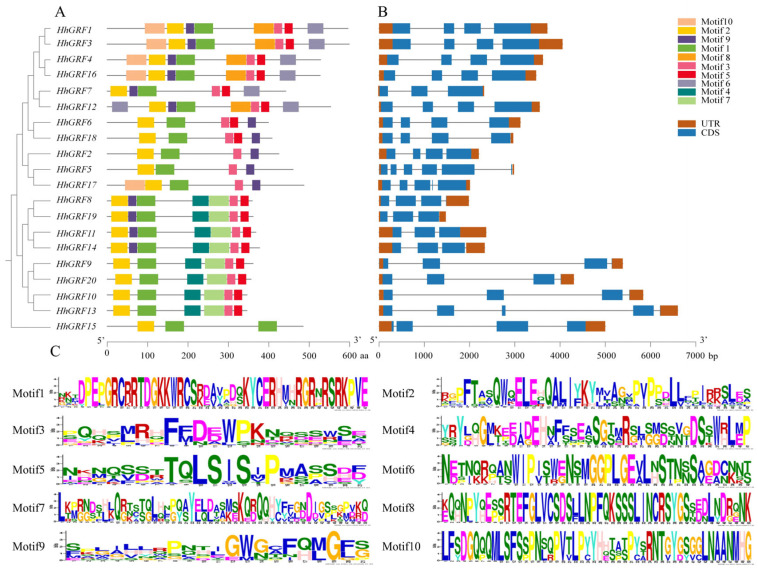
Conserved motifs (**A**), gene structure (**B**), and motif logo (**C**) of HhGRFs in *Hedera helix*.

Gene structure analysis determined that all members ranged from three to six coding sequences, with members of the same subfamily exhibiting nearly identical number and distribution position (Figure 5B). The conserved motif analysis showed remarkable consistency in motif composition and arrangement within subfamilies, with motif1 and motif2 being universally conserved across all 20 HhGRF members, while motif3 and motif5 displayed partial conservation, present in 19 and 16 members, respectively (Figure 5A). Sequence analysis of conserved motifs in HhGRF proteins revealed that motif1 and motif2 correspond to the characteristic WRC and QLQ domains of GRF proteins (Figure 5C, Table S2), providing molecular evidence for the fundamental structure conservation within GRF family. These findings collectively demonstrated both the evolutionary conservation and the subfamily specificity of HhGRF genes in *H. helix*.

### 2.8. Cis-Regulating Elements Analysis of HhGRF Gene Family

**Figure 6:**
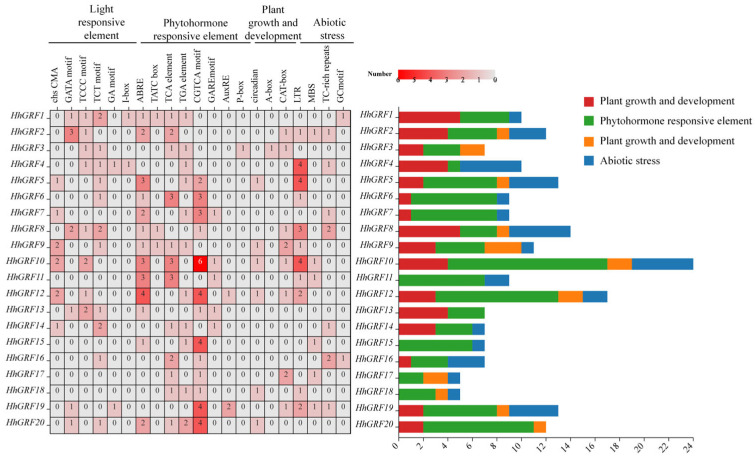
Cis-regulating element analysis of HhGRF genes in *Hedera helix*.

To elucidate the regulatory potential of HhGRF genes, a systematic analysis of cis-acting elements within their promoter regions was conducted. Twenty-one functionally distinct cis-elements were identified and categorized into four major classes: light responsive elements, phytohormone responsive elements, plant growth elements, and abiotic stress responsive elements (Figure 6). The number of phytohormone responsive elements predominated (total 208 elements), with the jasmonate-responsive CGTCA-motif being the most abundant (17% of total). It is worth noting that among the 20 HhGRF members, *HhGRF10* contained the highest number of cis-elements, particularly enriched in jasmonate-responsive elements. In contrast, *HhGRF17* and *HhGRF18* possessed the fewest cis-elements, while *HhGRF3* and *HhGRF20* completely lacked abiotic stress responsive elements (Figure S5). These findings demonstrated functional diversification among HhGRF family members in hormonal signaling and environmental adaptation.

### 2.9. Tissue-Specific Expression Levels of HhGRF Gene Family

**Figure 7:**
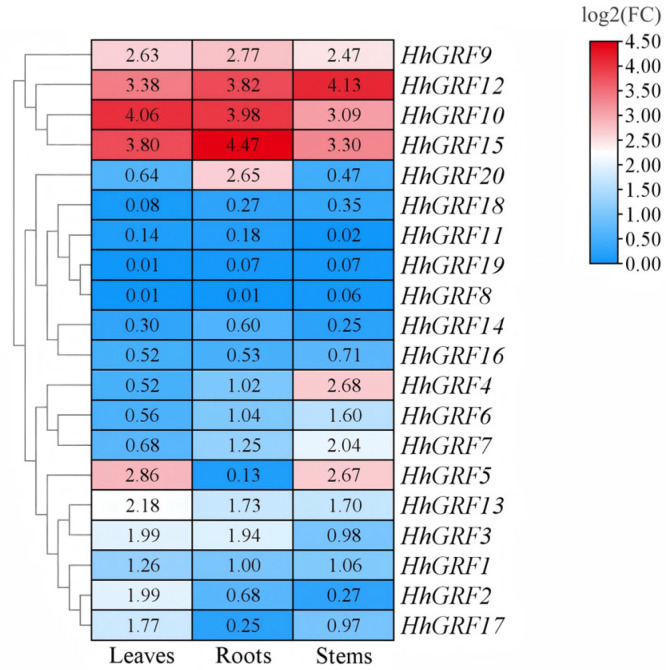
Tissue expression profiles of 20 HhGRF genes in *Hedera helix*.

Comprehensive qRT-PCR profiling of *HhGRF1*-*HhGRF20* expression in *H. helix* roots, stems, and leaves revealed striking tissue-specific expression patterns, as visualized by heatmap (Figure 7). *HhGRF10* displayed predominant expression in leaves compared to other genes, while *HhGRF15* showed root-specific accumulation, and *HhGRF12* exhibited stem-preferential expression. Notably, *HhGRF10*, *HhGRF15*, *HhGRF9*, and *HhGRF12* maintained consistently high expression levels across all examined tissues, suggesting their potential central roles in regulating fundamental growth processes in *H. helix.*

### 2.10. Gene Expression Analysis of HhGRF Gene Family Under MeJA Treatment

**Figure 8:**
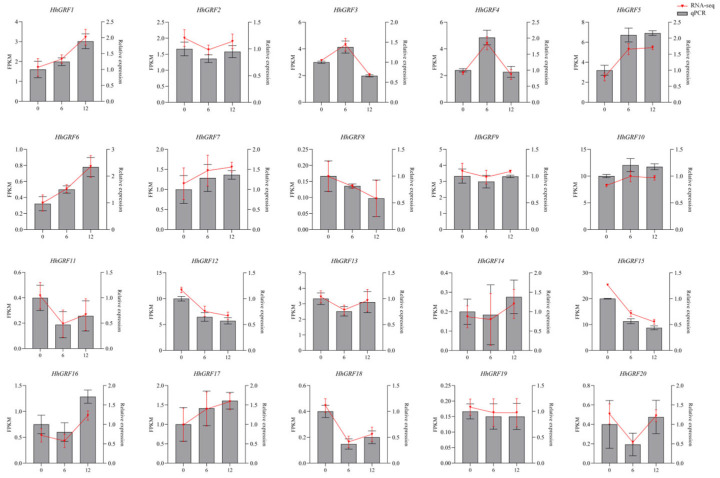
Expression profiles of 20 HhGRF genes under MeJA treatment for 0, 6, and 12 h in *Hedera helix*. Fragments Per Kilobase of transcript per Million mapped reads (FPKM) represents normalized gene expression levels in RNA-seq analyses.

Through an integrated approach combining qRT-PCR and RNA-seq analysis, the dynamic expression changes of *HhGRF1*-*HhGRF20* following MeJA treatment at 0, 6, and 12 h (Figure 8) were determined. The results demonstrated that MeJA treatment significantly modulated the transcription of HhGRF family members, with 8 genes (*HhGRF1*, *HhGRF3*, *HhGRF4*, *HhGRF5*, *HhGRF6*, *HhGRF7*, *HhGRF10*, and *HhGRF17*) showing markedly increased expression at 6 h post-treatment. Importantly, the expression peaks of *HhGRF1*, *HhGRF3*, *HhGRF4*, *HhGRF5*, *HhGRF6*, *HhGRF7*, *HhGRF10*, and *HhGRF17* were precisely similar with MeJA-induced flavonoid accumulation patterns, all peaking at 6 h before declining. Particularly, *HhGRF3*, *HhGRF4*, and *HhGRF10* exhibited strong positive correlations with flavonoid accumulation dynamics, strongly suggesting their regulatory roles in MeJA-induced flavonoid biosynthesis. Moreover, the high consistency between qRT-PCR and RNA-seq further validated the reliability of the results. This study provided the evidence for the crucial regulatory role of HhGRF transcription factors in connecting jasmonate signaling with flavonoid biosynthetic metabolism.

### 2.11. Transcriptional Regulation of Flavonoid Biosynthetic Enzymes in Response to MeJA Treatment

**Figure 9:**
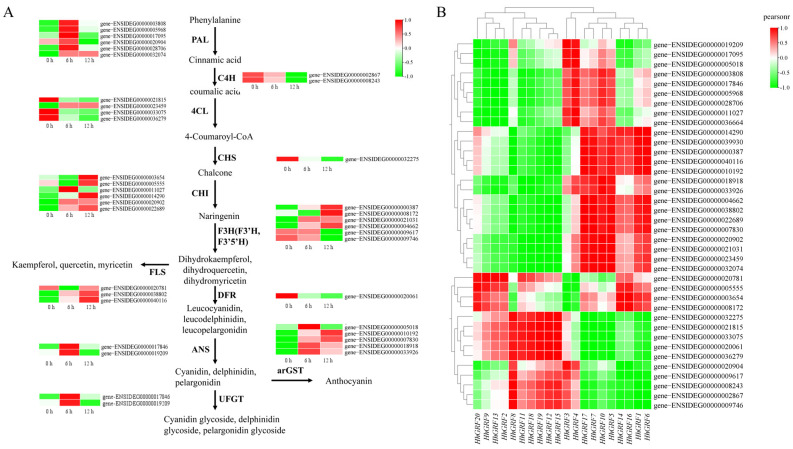
(**A**) Expression profiles of enzyme-encoding genes involved in flavonoid biosynthesis under MeJA treatment, (**B**) Correlation analysis of the expression between HhGRF genes and flavonoid biosynthetic genes.

In *H. helix*, several key enzymes involved in the phenylpropanoid pathway and flavonoid biosynthesis were identified (Table S3), such as PAL, C4H, and 4CL, which participate in the early steps of synthesizing the general phenylpropanoid. Additionally, enzymes like CHS and CHI, which play crucial roles in flavonoid accumulation, were detected. F3H is required for flavonoid production, while F3′H and F3′5′H mediate structural diversification through alternative hydroxylation patterns. The pathway also includes FLS, DFR, ANS, UFGT, and arGST, which are essential for the biosynthesis of various flavonoid derivatives; for example, arGST can catalyze an essential dehydration of the leucoanthocyanidin dioxygenase product to generate cyanidin [9]. Using the KIPEs3 tool, the key flavonoid biosynthesis enzymes of *H. helix* were identified. Finally, 38 putative key enzymes were found. Moreover, the FPKM of 38 key enzymes following MeJA treatment at 0 h, 6 h, and 12 h was analyzed (Figure 9A, Table S4).

The majority of key enzymes (24/38) exhibited upregulated expression at 6 h post-MeJA treatment, suggesting their potential functional significance in flavonoid biosynthesis in *H. helix*. 15 genes displayed a characteristic biphasic expression pattern (initial increase followed by decline), which showed strong positive correlation with both the expression profiles of three key HhGRF genes (*HhGRF3*, *HhGRF4*, and *HhGRF10*) and the dynamic accumulation patterns of flavonoids in *H. helix* leaves. Notably, the early phenylpropanoid pathway genes, such as PAL (all) and 4CL (1/4), displayed more rapid induction (6 h), while downstream flavonoid biosynthetic enzymes, such as CHI (4/6), F3H (3/6), and FLS (2/3), showed maximal expression at 12 h, suggesting the sequential transcriptional activation of the metabolic pathway. Moreover, *HhGRF1*-*HhGRF20* exhibits a significant correlation with key flavonoid biosynthetic enzymes (Figure 9B). Importantly, combined with its tissue-specific expression pattern, *HhGRF10* emerges as a particularly strong candidate regulator of MeJA-induced flavonoid accumulation in *H. helix* leaves.

### 2.12. The Overexpression of HhGRF10 Promotes the Accumulation of Flavonoids in H. helix

**Figure 10:**
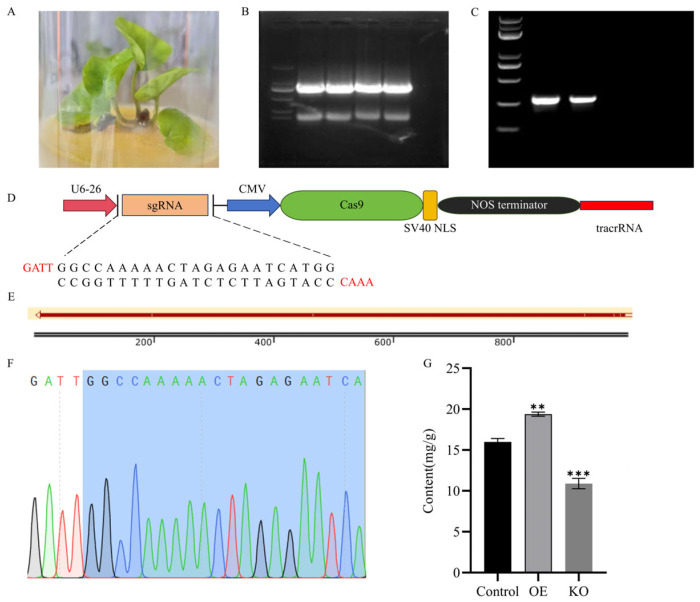
(**A**) Transient transformation of *Hedera helix*, (**B**) Vector construction for OE-*HhGRF10*, (**C**) Vector construction for KO-*HhGRF10*, (**D**) Gene knockout strategy of *HhGRF10*, (**E**) Sequencing alignment of OE-*HhGRF10*, (**F**) Target site sequencing of KO-*HhGRF10*, (**G**) The flavonoid content in the control, overexpression (OE), and knockout (KO) lines. Significant differences: ** (*p* < 0.01), and *** (*p* < 0.001). Different colored fonts represent different bases.

To investigate the function of *HhGRF10* in flavonoid accumulation, we generated both overexpression (OE) and knockout (KO) constructs and transformed them into *Agrobacterium tumefaciens* GV3101. Transgenic *H. helix* plants were obtained through transient transformation, with successful transformation confirmed by PCR-based genotyping (Figure S6). Subsequent content analysis revealed significant alterations in flavonoid accumulation across transgenic lines (Figure 10G): OE-*HhGRF10* plants exhibited a 1.2-fold increase in total flavonoid content compared to control, whereas KO lines showed a 32% reduction. These results provide direct genetic evidence that *HhGRF10* positively regulates flavonoid biosynthesis in *H. helix*.

## 3. Discussion

Flavonoids represent a major class of polyphenolic secondary metabolites that predominantly accumulate as glycosides in medicinal plants, exhibiting diverse biological functions including potent antioxidant activity [34]. While traditional research has focused on extraction methodologies and pharmacological properties, contemporary investigations increasingly emphasize elucidating the mechanisms governing flavonoid accumulation. In *H. helix*, flavonoids primarily accumulate in leaf tissues, where MeJA treatment serves as a critical elicitor of specialized metabolite production. As a potent exogenous elicitor and signaling molecule, MeJA significantly enhances specialized metabolite accumulation in medicinal plants. In *Platycodon grandiflorus*, MeJA enhances saponin accumulation by upregulating the transcription factor PgbHLH28, which subsequently activates key biosynthetic genes (*PgHMGR2* and *PgDXS2*) in the saponin pathway [35]. In *Dioscorea composita*, MeJA promotes proanthocyanidin accumulation by upregulating the expression of *DcWRKY11*, which directly binds to the promoter region of *TT2* (a key proanthocyanidin biosynthetic regulator) to activate downstream gene expression [36]. Similar to other species, *H. helix* exhibits significant responsiveness to MeJA treatment, effectively stimulating the biosynthesis of specialized metabolites. Our experimental treatment with 100 μM MeJA demonstrated a significant induction of flavonoid accumulation in *H. helix* leaves, with peak levels observed at 6 h post-treatment.

Advancements in high-throughput transcriptomic technologies have facilitated large-scale gene expression analyses, enabling systematic analyses of candidate genes involved in flavonoid biosynthesis pathways. Comparative transcriptome has provided vital insights into the evolutionary conservation and species-specific flavonoid biosynthesis of plants such as *Citrus reticulata* [37], and *P. quinquefolius* [38]. Herein, we performed RNA-seq of MeJA-treated *H. helix* leaves to elucidate MeJA-induced flavonoid accumulation. Transcriptomic profiling revealed a time-dependent increase in DEGs across treatment durations, with 28,202, 29,723, and 30,772 DEGs identified at 0, 6, and 12 h respectively. This progressive transcriptional process reflects the activation of complex adaptive mechanisms in response to hormone stimulation. Functional enrichment and pathway analyses provided partly mechanistic insights into MeJA-induced flavonoid biosynthesis in *H. helix*. GO enrichment revealed not only the activation of signing-response pathways but also a coordinated upregulation of specialized metabolic processes, particularly flavonoid biosynthesis, suggesting a targeted reprogramming of secondary metabolism. The significant enrichment of lipid metabolic processes may reflect the requirements for phenylpropanoid synthesis. KEGG analysis pinpointed the phenylpropanoid biosynthesis pathway (ko00940) as the most significantly enriched, with 12 key enzymes being differentially expressed, including *PAL*, *4CL*, and *CHS*, which collectively explain the observed flavonoid accumulation patterns. This systematic activation from upstream signaling through to metabolite synthesis demonstrates the ability of MeJA to precisely modulate the flavonoid biosynthetic machinery in *H. helix*.

Our transcriptome analysis of MeJA treated in *H*. *helix* revealed the significant modulation of a GRF transcription factor family, suggesting their potential involvement in jasmonate-mediated responses. The GRF transcription factors, known to coordinate plant development and stress responses, was systematically characterized in *H. helix* in this study. Our genome-wide analysis identified 20 GRF family members, more than *A. thaliana* (9), *O. sativa* (12), or *Z. mays* (15) but fewer than *N. tabacum* (25) or *G*. *max* (22). This lineage-specific gene family expansion likely results from evolutionary genomic duplication events, potentially conferring enhanced adaptive capacity in *H. helix* [39]. A phylogenetic tree classified the HhGRF proteins into five distinct groups, each demonstrating conserved structure features and motif characteristics. Notably, comparative analysis revealed closer evolutionary relationships between HhGRFs and eudicot orthologs (particularly *A. thaliana*) than with monocot representatives, supported by both phylogenetic clustering and synteny analysis. Two particularly noteworthy evolutionary patterns emerged from our analysis. The Group II GRFs, while absent in *A. thaliana*, were retained in *H. helix*, suggesting potential neofunctionalization events specific to this lineage. Moreover, the Group IV members appear to represent a eudicot conserved GRF subfamily that has been selectively lost in monocot species. These findings collectively illustrate how the differential retention and diversification of GRF family members may contribute to species-specific adaptations in *H. helix*.

GRFs are increasingly recognized as key mediators of hormone signaling pathways. In *Saccharum spontaneum*, SsGRF7 promotes leaf elongation through auxin (IAA) pathway activation [40]. AtGRF5 interacts with DELLA proteins to enhance cold stress tolerance via gibberellin-mediated signaling in *A. thaliana* [41]. Tea plant (*Camellia sinensis*) GRFs respond to multiple phytohormones including GA3, IAA, SA, ABA, and MeJA to coordinate leaf development [42]. These regulatory effects are achieved through the direct binding of GRFs to specific cis-elements in target gene promoters. Our cis-element analysis identified abundant jasmonate-responsive elements in HhGRF promoters, implying their potential involvement in MeJA signaling. This was further supported by the dynamic expression changes of HhGRF members (particularly *HhGRF1*, *HhGRF3*, *HhGRF4*, *HhGRF5*, *HhGRF6*, *HhGRF7*, *HhGRF10*, *HhGRF17*) following MeJA treatment, these eight genes showing significant upregulation at 6 h post-treatment. Notably, the biphasic expression patterns of *HhGRF3*, *HhGRF4*, and *HhGRF10* precisely mirrored the temporal accumulation profile of flavonoids, strongly suggesting their regulatory roles in MeJA-induced flavonoid biosynthesis.

The coordinated induction of 31 flavonoid pathway enzymes following MeJA treatment, including 13 genes exhibiting expression patterns synchronous with *HhGRF3*, *HhGRF4*, and *HhGRF10*, further supports this regulatory network. In particular, eight key enzymes showed sequential activation peaking at 6 h, coinciding with the expression of *HhGRF10*. These findings collectively position *HhGRF10* as a master regulator that potentially activates flavonoid biosynthetic genes to promote flavonoid accumulation in *H. helix* leaves. Our findings demonstrate that *HhGRF10* overexpression significantly increased flavonoid content in *H. helix* whereas the knockout of *HhGRF10* reduced flavonoid accumulation, unequivocally confirming the pivotal role of *HhGRF10* in regulating flavonoid biosynthesis in *H. helix*. Furthermore, tissue-specific expression analysis in *H. helix* revealed that *HhGRF10*, *HhGRF15*, *HhGRF9*, and *HhGRF12* maintain consistently high expression levels across all examined tissues, with *HhGRF10* showing a leaf-predominant expression, suggesting that *HhGRF10* dually regulates both flavonoid accumulation and plant growth in *H. helix*. The evolutionary conservation of this GRF-flavonoid module across diverse species, coupled with its unique features in *H. helix*, offers new insights into the molecular mechanisms underlying specialized metabolism in lianas medicinal plants.

## 4. Materials and Methods

### 4.1. Plant Growth and Experimental Treatments in H. helix

Healthy, uniformly grown *H*. *helix* seedlings were cultivated in Murashige and Skoog (MS) medium supplemented with 2 mg·L^−1^ 6-benzyladenine (6-BA, Aladdin, Shanghai, China), 0.5 mg·L^−1^ 1-naphthaleneacetic acid (NAA, Aladdin), 5% sucrose, and 0.8% agar under controlled conditions at 25 ± 1 °C and 65% relative humidity, a cycle of 16 h light and 8 h dark for 8 weeks [43]. The *H. helix* seedlings were identified by Professor Shen Xiaoxia (Zhejiang Chinese Medicine University). Different tissues (Roots, leaves, and stems) were obtained for tissue-specific expression analysis. For MeJA induction, to investigate the effect of hormones on the expression level of HhGRF genes, *H. helix* seedlings were evenly sprayed with 100 μM MeJA, while control plants received water. Plant samples were collected at 0 h, 6 h, and 12 h post-treatment, with three biological replicates at each time point. All samples were immediately frozen in liquid nitrogen and stored at −80 °C until analysis.

### 4.2. Phytochemical Content Analysis of Flavonoids in H. helix

Total flavonoid content was quantified using an optimized NaNO_2_-Al(NO_3_)_3_-NaOH protocol [44]. Briefly, 0.1 g tissue was extracted with 1 mL 80% methanol (HPLC grade, Agilent, Hangzhou, China) using ultrasonic-assisted extraction (80 kHz, 60 min). After centrifugation (12,000 rpm, 10 min), the 0.5 mL supernatant was reacted sequentially with 0.3 mL 5% NaNO_2_ (*w*/*v*), 0.3 mL 10% Al(NO_3_)_3_ (*w*/*v*), and 3mL 1 M NaOH. Absorbance was measured at 510 nm using a UV/Vis spectrophotometer (Perkinelmer Lambda 365, Waltham, MA, USA), with rutin as the standard. Method validation confirmed linearity (R^2^ = 0.9995), yielding the linear equation as y = 0.1413x+ 0.0411. 

### 4.3. Transcriptome Profiling and Bioinformatics Analysis

RNA extraction was performed with the RNA Isolation Kit (Huayueyang Biotechnology, Beijing), according to the manufacturer’s protocol. RNA quality was assessed on a spectrophotometric Nanodrop 2000 system (Thermo Fisher, Waltham, MA, USA). RNA-seq libraries were carried out prepared from 1 μg total RNA using poly(A) selection with oligo(dT) beads (Thermo Fisher). Subsequently, mRNA was isolated and fragmented using the U-mRNAseq Library Prep Kit (Kaitai-bio, Hangzhou, China). Fragmented mRNA was reverse-transcribed into cDNA by the SMARTScribe Kit (Takara Bio., Beijing, China), followed by end repair, A-tailing, Illumina adapter ligation, and PCR amplification (15 cycles). Qualified libraries were proportionally pooled for 150 bp paired-end sequencing on the Illumina NovaSeq 6000 platform, generating approximately 40 million reads per sample. To ensure data quality for analyses, we removed raw sequencing reads containing >5% ambiguous bases (N), discarding reads where the number of bases with Q ≤ 10 accounts for more than 50% of the entire Read, and removing regions with Phred quality scores below 10. The transcriptomic analysis was conducted on the BMKCloud platform (https://www.biocloud.net/, accessed on 6 May 2025), including HISAT2 v2.2.1 (alignment to reference genome) [45], StringTie v2.1.4 (transcript assembly) [46], featureCounts v2.0.1 (read quantification) [47], and DESeq2 v4.11 (DEGs analysis) [48]; all parameters remained default.

### 4.4. PCA Analysis and Differentially Expressed Genes Identification

PCA was performed on variance-stabilized transformed counts using the DESeq2 in BMKCloud platform. For differential expression analysis, we implemented a negative binomial generalized linear model specifically designed for RNA-seq count data. Statistical significance thresholds were set at a minimum fold change of 1.2 with a false discovery rate (FDR) below 0.01, applying the Benjamini–Hochberg procedure to control for multiple testing.

### 4.5. Functional Enrichment and Pathway Analysis

GO term enrichment analysis was carried out utilizing the web-based platform Enrichr (https://maayanlab.cloud/Enrichr/, accessed on 6 May 2025). KEGG pathway enrichment was executed employing the KOBAS software (version 2.8.3) in the BMKCloud platform [49], with statistical significance assessed through hypergeometric testing followed by false discovery rate correction (FDR < 0.05). 

### 4.6. Systematic Identification of GRF Gene Family Members in H. helix

The nine GRF proteins in *A. thaliana* were obtained from the TAIR database (https://www.arabidopsis.org/, accessed on 5 May 2025) as queries for BLAST searches in the genome of *H. helix* to obtain the GRF members with the TBtools software (v2.119) (E-value < 1 × e^−5^) [50]. Two HMM Models of the QLQ domain (PF08879) and the WRC domain (PF08880) were recruited from the PFAM (http://pfam.xfam.org/, accessed on 13 April 2025), and target hits within the QLQ and WRC domains were identified using GRF members through TBtools.

Then, these sequences were submitted to SMART (https://smart.embl.de/, accessed on 13 April 2025) to verify these conserved domains. Subsequently, they were named *HhGRF1*-*HhGRF20*.

### 4.7. Phylogenetic Tree Construction of GRF Family Proteins

Complete GRF protein sequences of *A. thaliana* and *O. sativa* were retrieved from the TAIR database and Phytozome database (https://phytozome.jgi.doe.gov; accessed 6 May 2025) (Table S5). These sequences were subjected to multiple sequence alignment using the DNAMAN 9.0 software (https://www.lynnon.com/dnaman.html; accessed on 8 June 2025) with default parameters. Molecular phylogenetic analysis was subsequently performed through the maximum likelihood (ML) method implemented in MEGA6 [51], with statistical support assessed by 1000 bootstrap replicates.

### 4.8. Chromosome Localization Multiple Species and Collinearity Analysis of HhGRF

The chromosomal locations of all HhGRF genes were precisely mapped based on the *H. helix* genome annotation using TBtools. For evolutionary analysis, we employed a multi-step approach to identify gene duplication events and syntenic relationships using the Advanced Circos in TBtools. Additionally, we performed comparative synteny analysis between *H. helix* and *A. thaliana*, *O. sativa* to identify conserved regions in TBtools. 

### 4.9. Exon-Intron Arrangement, Conserved Motifs, and Cis-Regulatory Element in HhGRF Genes

Conserved motifs were identified using the MEME website (v5.5.6) (https://meme-suite.org/, accessed on 18 April 2025) with these parameters: maximum 10 motifs, width 6–50 aa, E-value < 1 × e^−10^. Gene structures were annotated using TBtools to explore the distribution of exons and introns. The promoters of HhGRF were extracted from the genome with the GFF3 Sequence Extract and Fasta Extract or Filter of the TBtools software, and they were then uploaded to PlantCARE (https://bioinformatics.psb.ugent.be/webtools/plantcare/html/, accessed on 19 April 2025) to predict the cis-regulatory element. 

### 4.10. Total RNA Extraction and qPCR Analysis

RNA isolation was performed according to the methodology detailed in Section 4.3, and then the total RNA (1 μg) was reverse-transcribed into cDNA using the Evo M-MLV RT Kit II (Accurate Biology, Changsha, China) to acquire cDNA for detecting the expression level of the genes. The qRT-PCR primers were designed by PrimerQuest website tool (https://sg.idtdna.com/PrimerQuest/Home/Index, accessed on 25 April 2025) and generated in Zhejiang Sunya Biotechnology company (Hangzhou, China) (Table S6). The reaction mixture of qRT-PCR was 5 μL SYBR Green Mix (Accurate Biology), 1 μL cDNA, 0.4 μL of each primer (10 μM), and 3.2 μL RNase-free water; qPCR was performed with an initial denaturation (95 °C, 30 s) followed by 40 cycles of denaturation (95 °C, 5 s), annealing (55 °C, 30 s), and extension (72 °C, 30 s), and reaction on a fluorescence quantitative PCR (Life ABI7500, Waltham, MA, USA). Additionally, the 2^−ΔΔCt^ method was employed to calculate the relative expressions [52], and three different β-actin genes were selected as the reference control based on its stable expression [53].

### 4.11. Identification of Key Enzymes Involved in Flavonoid Biosynthesis of H. helix

The protein sequences were analyzed with KIPEs3 v3.2.6 [54] to identify flavonoid biosynthesis genes in *H. helix*. The bait sequences and the flavonoid biosynthesis dataset (v3.3) of KIPEs3, PAL, C4H, 4CL, CHS, CHI, F3H, FLS, DFR, ANS, and UFGT were identified. Following this, the identification of arGST proteins was performed through the GST database of KIPEs3 (v3.3).

### 4.12. Molecular Cloning, Vector Construction and of Transgenic Plants Generation

The *HhGRF10* overexpression vector was constructed by amplifying the target gene using PrimeSTAR Max DNA Polymerase (Takara Bio., Beijing, China), followed by cloning into pCAMBIA1301 (Cambia, Canberra, Australia). The primers for knockout (KO) *HhGRF10* were designed on the CRISPRdirect server (https://crispr.dbcls.jp/, accessed on 8 June 2025) (Table S7). The synthesized sgRNA fragments were subsequently inserted into a Cas9-expression vector and finally assembled into pCAMBIA2300 (Cambia).

The *HhGRF10* overexpression (OE) and knockout (KO) constructs were introduced into *A. tumefaciens* strain GV3101 (Weidi Biotechnology Co., Shanghai, China) via the freeze–thaw method. The *Agrobacterium* cells were resuspended in infiltration buffer containing 10 mM MES (Aladdin), 100 μM acetosyringone (Aladdin), and 0.1% (*v*/*v*) Tween 20 (Aladdin). The transient transformation of *H. helix* was performed using *Agrobacterium*-mediated infiltration. All experiments were performed using T3 generation plants to ensure a stable genetic background. Five biological replicates for each line were conducted to avoid potential variation. Putative transgenic plants were screened by PCR amplification.

## 5. Conclusions

This study conducted a systematic analysis of the transcriptome of *H*. *helix* treated with MeJA. Transcriptome analysis revealed dynamic gene expression changes under MeJA treatment, with DEGs significantly enriched in hormone signaling as well as phenylpropanoid metabolism pathways. We identified 20 HhGRF genes phylogenetically clustered into five distinct groups, showing closer evolutionary relationships with eudicots than monocots. HhGRFs exhibited tissue-specific expression patterns and responsiveness to jasmonate signaling, with *HhGRF10* emerging as a key regulator due to its constitutive high expression across tissues and strong correlation with flavonoid accumulation dynamics. The coordinated induction of *HhGRF10* and flavonoid biosynthetic enzymes following MeJA treatment suggests its dual role in regulating both flavonoid production and leaf development. The overexpression of *HhGRF10* significantly enhanced flavonoid accumulation in *Hedera helix*, demonstrating its crucial role in regulating flavonoid biosynthesis. These discoveries provide a critical mechanistic understanding of flavonoid biosynthesis in *H. helix* and establish HhGRFs, particularly *HhGRF10*, as promising targets for metabolic engineering to enhance bioactive compound production in this medicinally important species.

## Data Availability

All data generated or analyzed in this study are included in the main text and its Supplementary Materials.

## Supplementary Materials

The following supporting information can be downloaded at: https://www.mdpi.com/article/10.3390/plants14142094/s1, Table S1: Summary of RNA-Seq data in *H. helix* after MeJA treatment; Table S2: The information of motif 1–10 using MEME server; Table S3: The protein sequences of the enzymes related to flavonoid biosynthesis in *H. helix*; Table S4: The FPKM value of HhGRFs and enzymes related to flavonoid biosynthesis through RNA-seq technology under MeJA treatment; Table S5: The protein sequences of GRF family in *H. helix*, *A. thaliana*, *O. sativa;* Table S6: Primers used for quantitative real-time PCR; Table S7: Primers used for molecular cloning and vector construction. Figure S1: Principal component analysis (PCA) of all samples; Figure S2: Gene Ontology (GO) enrichment analysis of DEGs; Figure S3: Analysis of differentially expressed transcription factors; Figure S4: Chromosomal localization of GRF genes in *H. helix*; Figure S5: The positions and numbers of cis-regulating element of HhGRF genes in *H. helix*; Figure S6: Positive PCR identification of OE (A) and KO (B) in transgenic plants.
