# Supplementary material for: Genome-Wide Transcriptome Analysis Reveals GRF Transcription Factors Involved in Methyl Jasmonate-Induced Flavonoid Biosynthesis in Hedera helix
Source: Plants (Basel). 2025 Jul 8;14(14):2094. doi: 10.3390/plants14142094 (PMC12298800; doi:10.3390/plants14142094)
Supplement: Supplementary file 1 [file plants-14-02094-s001.zip › plants-3686962-supplementary.pdf]

Supplementary Materials

Table legends

Table S1 Summary of RNA-Seq data in *H. helix* after MeJA treatment.

Table S2 The information of motif 1-10 using MEME server.

Table S3 The protein sequences of the enzymes related to flavonoid biosynthesis in *H. helix*.

Table S4 The FPKM value of HhGRFs and enzymes related to flavonoid biosynthesis through RNA-seq technology under MeJA treatment.

Table S5 The protein sequences of GRF family in *H. helix, A. thaliana,* and *O. sativa.*

Table S6 Primers used for quantitative real-time PCR.

Table S7 Primers used for molecular cloning and vector construction.

Figure legends

Figure S1 Principal component analysis (PCA) of all samples.

Figure S2 Gene Ontology (GO) enrichment analysis of DEGs.

Figure S3 Analysis of differentially expressed transcription factors.

Figure S4 Chromosomal localization of GRF genes in *H. helix*.

Figure S5 The positions and numbers of cis-regulating element of HhGRF genes in *H. helix.*

Figure S6 Positive PCR identification of OE (A) and KO (B) in transgenic plants.

Table S1 Summary of RNA-Seq data in *H. helix* after MeJA treatment

**Samples**	**Clean reads**	**GC Content**	**%≥Q30**
M0-1	23,026,217	43.39%	93.97%
M0-2	23,099,344	43.40%	92.90%
M0-3	23,159,228	43.99%	93.67%
M6-1	23,949,309	43.16%	93.42%
M6-2	23,168,680	43.13%	93.59%
M6-3	24,300,570	43.65%	92.85%
M12-1	23,694,562	43.30%	93.67%
M12-2	23,790,457	42.90%	93.32%
M12-3	23,759,677	42.71%	93.39%

Table S2 The information of motif 1-10 using MEME server.

Motif	Name	E-value	Sites	Width
Motif 1	NKTDPEPGRCRRTDGKKWRCSRDAVPDQKYC ERHMHRGRNRSRKPVE	$1.3e^{-816}$	20	47
Motif 2	RGPFTASQWQELEHQALIYKYMVAGVPVPPDL LFPIRRSLES	$7.8e^{-483}$	20	42
Motif 3	PQHSLRHFFDEWPKNQSSWSE	$4.9e^{-141}$	19	21
Motif 4	YRYLHGLKEEIDEHNFFSEASGSMRSLGGDSVG DSSWRLEP	$2.3e^{-122}$	8	41
Motif 5	NDNQSSTTQLSISIPMASSDF	$3.2e^{-112}$	16	21
Motif 6	NETNQRQANWIPISWENSMGGPLGEVLHSTN NSAGDCKNS	$1.4e^{-088}$	6	40
Motif 7	LKPRNDSHLQRTSTQLHLPQAYELDAAMSKQR QQHYFFGBDIGSPGPVKQ	$7.4e^{-098}$	7	50
Motif 8	EQQNPYQEPSRTEFGLVCSDSLLNPFQKSSSLIN CRSYGSSDDLNDRQNK	$5.1e^{-072}$	4	50
Motif 9	SPLALHRPNTJGWGAFQMGFS	$3.9e^{-071}$	14	21
Motif 10	LFSDGQQQMLSFSSPNLQPVTLPYYHHSASPYS RNTGYGSGGLNAANMHG	$3.3e^{-069}$	4	50

Table S3 The protein sequences of the enzymes related to flavonoid biosynthesis in *H. helix*.

Name	Protein sequences
>gene-ENSIDEG00000000387	MAQTTTVRSFLQVAATEEVASPLRVVQIEGLAILKIIKH<br>CKEFSPALVTGQLLGLDVGSVLEVTNCFPFPIREEDEEIE<br>AEGANYQLEMMRCLREVNVDNNTVGWYQSTLLGSY<br>QTVELIETFMNYQENIKRCVCIIYDPSKSNQGVLALKAL<br>KLSDSFMELYRSKNFTGEKLREKNLSWVDIFEEIPIKVSN<br>SALISAFMTELEADSPVTQCDYDRLQLSTNPFMERNVE<br>FLIECMDDLSMEQQKFQFYYRNLSRQQAQQQAWLQK<br>RRAENMARKAAGEEPLPEEDPSNPVFKPIQEPPRLDSF<br>LITNQISNYCNQINGVAGQSFSKLYLMKALHEN
>gene-ENSIDEG00000002867	MDLLLLEKGLVALFFAVIVAISISKLRGKRFKLPPGPIPVP<br>VFGNWLQVGDDLNHRNLTDYAKKFGDVFLLRMGQR<br>NLVVVSSPDHAKEVLHTQGIEFGSRTRNVVFDIFTGKG<br>QDMVFTVYGEHWRKMRRIMTVPFFTNKVVQQYRHG<br>WEDEVAHVVADVKANPEASTTGIVLRRRLQLMMYNM<br>MYRIMFDRRFESEEDPLFLKLKALNGERSRLAQSFGYN<br>YGDFIPILRPFLRGYLKICKEVKEKRLQLFKDYFVDERKK<br>LESTKRMDNNSLKCAMDHILEAQQKGEINEDNVLYIV<br>ENINVAAIETTLWSIEWGIAELVNHPEIQQKLRHEIDTV<br>LGPGVQVTEPDTHKLPYLQAVIKETLRLRMAIPLLVPH<br>MNLHDAKLGSYDIPAESKILVNAWWLANNPAHWKYP<br>EEFRPERFVEEESKVEANGNDFRYLPFGVGRRSCPGIIL<br>ALPILGITLGRLVQNFELLPPPGQPKIDTAEKGGQFSLHI<br>LKHSTIVAKPRSVS
>gene-ENSIDEG00000003654	MGLMATDFFVGLCVLILGICFVNGRFVVEKSSISVISPYD<br>LRSEKHDSAIGNFGVPDYGGSMVGSVVYPHKGSYGCA<br>AFDGDKPFKSKSSRPSILLLDRGECYFALKVWNGQQAG<br>AAAVLVADSRDEPLITMESPEESSDADGYIDKIGIPSALI<br>NRAFGETLKAAIQKGEEDVVIKLDWRESMPHPDQRVE<br>YEFWTNSNDECGIRCNEQMNFVKDFKGHAQILEKGG<br>YTLFTPHYITWYCPRPFILSNQCKSQCINHGRYCAPDPE<br>KDFGEGYQGKDVVFENLRQLCVHRVANESNRSWVW<br>WDYVTDFHIRCSMKEKRYSKECAEDVMKSLNLPIEKIK<br>GCMGDPEADVENEVLKIEQDRQIGRGSRGDVTILPTLVI<br>NDVQYRGKLDRTAVLKAICAGFQETAEPSICLSGDLET<br>NECLERNGGCWRDSQSNITACKDTFRGRVCECPVANG<br>VQYKGDGYKSCEAFGPGRCAINNGGCWSETKYGQTLS<br>ACSDSDISGCRCPPGFRGVGSKCEDINECTEDLVCQCE<br>GCSCKNTWGGYGCKCKGDKLYIMEQDTCIERNTSKFG<br>WFLTLLILGAVVTAATAGYIFYKYRLRSYMDSEIMAIMS<br>QYMPLDSQHHNQVVQHEGEPLRQGSSSV
>gene-ENSIDEG00000003808	MSRFQPTPPASHPTLFANGETYLPRQNGNGHALLDSSC<br>PKDPLNWGLAADSLRGSHVDEVKRMVGEFRKPVVRL

GGESLTVAQVAAVAGGGGGVVVELCEAARGGVKASSE
WVMESMNKGTDSYGVTTGFGATSHRRTKQGAALQKE
LIRFLNAGIFDNGTESSHRLPHSATRAAMLVRINTLLQG
YSGIRFEILEAITKLLNHNITPCLPLRGTITASGDLVPLSYI
AGLLTGRPNSKCMGPTGKSLDPGKAFRLAGIEGGFFEL
QPKEGLALVNGTAVGSGLASTVLFETNVLALLGEVLSA
VFAEVMQGKPEFTDHLTHKLKHHPGQIEAAAIMEHIL
DGSSYVKAAKKLHEMDPLQKPKQDRYALRTSPQWLG
PQIEVIRASTKSIEREINSVNDNPLIDVSRNKALHGGNF
QGTPIGVSMDNTRLAIAAIGKLMFAQFSELVNDFYNNG
LPSNLSGGRNPSLDYGFKGAEIAMAAYCSELQFLANPV
TNHVQSAEQHNQDVNSLGLISSRKTAEAVDILKLMSST
YLVALCQAIDLRHLEENMKNTVKNTVSQVAKKVLIMG
LNGELQNSKFCEKDLLKEVDREHVFAYIDDPCSATYPL
MQKLRQVLVDHALINSEDSKNLGTSIFLKIGSFEEELMT
LLPKEVESTRSAFENGNLQIPNRIRECRSYPLYKFVRDDL
GGEYLTGEKIRSPGEEFDKVFTAICEGKIIDPLLQCLKD
WDGAPLPIC

>gene-ENSIDEG00000004662 METLPIWAAYTAFATLALFLLSKYLRPNLNTPPGPKPWP
IIGNLNLIGSLPHRSIHQLSRQYGPIMQLRFGSCPVVVGS
SAEMAKIFLKTMDVNFVGRPKTAAGKYTTYNYSDITW
SPYGPYWRQARRICLMELFSAKRLESYEYIRVEEMKSML
KLLYGLSGQKIALKDYLSTVSLNVISRMVLGKRYLDESS
VEKAIVKPDEFKKMLDELFLLNGVFNIGDSIPWIDFLDL
QGYVKRMKVVSKKFDRFLEHVLNEHIARRENEKGDYV
AKDMVDLLLQLSDDPTLEVKLERHGVKAFTQDLLAGG
TESSAVTVEWAISQLLKKPEIFEKATEELDRVIGKNRWV
EEKDIQNLPYIQAIVKETMRLHPVAPMLVPRVARVDCK
VASYDIVKGSRILVSVWTIGRDPTLWDEPDEFVPKRFIG
KTTDVKGHDFELLPFGAGRRMCPGYTLGLKVIESSLAN
LLHGFNWKLPDSMTSEDLDMDEIFGLSTPKKIPLVTIAE
PRLPLELYSL

>gene-ENSIDEG00000005555 MEKLGFFVCVWFLLYGSCLGRFVVEKNSLKVTSPDNLK
DTYECAIGNFGVPQYGGTLVGAVIYPKANQKACKSFTD
VDISFKSKPGGLPIFLLADRGDCYFTLKAWNAQMAGA
AAILVADDRVEPLITMDTPEEEDARADYLQNITIPSALIS
KSLGDKIKKALSSGDMVNINLDWRESLPHPDERVEYEF
WTNSNDECGPKCESQIEFVKNFKGVAQILERKGYTQFT
PHYITWYCPEAFILSKQCKSQCINHGRYCAPDPDQDFS
KGYNGKDVVVQNLHQACFYKVANESGKPWLWWDYV
TDFAIRCPMKDKKYTTECADQVIQSLGVDLKQIDKCVG
DPNADIDNPILKIEQEAQIGKGSRGDVTILPTLVINNRQ
YRGKLDKGAVLKAICSGFEEKTEPAICLSEGIQTNECLE
NNGGCWQDKAANVTACKDTFRGRVCECPIVQGVKFS

GDGYTHCEASGALRCEINNGGCWRKTQNGRTYSACID
DHTKGCKCPPGFKGDGVNSCEDIDECKEKLACQCSEC
KCKNTWGSYECSCSGNLLYMREHDTCISKDVNAEVSW
GFVWVIILGLAAAGVGGYAMYKYRIRRYMDSEIRAIMA
QYMPLDNQPEVPNHLSQGSV

>gene-ENSIDEG00000005968 SHLSRSSAVRSEERSNGHDTIPSSNSTHSFPPNSLSTSLFF
NYSHNFLSHPKIIRKPKIPLALAVNGMELPHQNGNGH
VLLDGLCAKDPLNWGLAVDSLRGSHVDEVKRMVGEF
RKAVVRLGGESLTVAQVAAVAAGGGRMVVELCEAAR
GGVKASSEWVMESMNKGTDSYGVTTGFGATSHRRTN
QGAALQKELIRFLNAGIFGNGTESSHTLPHSATRAAML
VRINTLLQGYSGIRFEILEAITKLLNHNITPCLPLRGTITA
SGDLVPLSYIAGLLTGRPNSKCVGPTGESLDVRKAFCLA
GIEGGFFELQPKEGLALVNGTAVGSGLASVVLFETNILA
LLAEVLSAVFAEVMQGKPEFTDHLTHKLKHHPGQIEA
AAIMEHILDGSSYVKTAKKLHEMDPLQKPKQDRYALR
TSPQWLGPQIEVIRASTKSIEREINSVNDNPLIDVSRNKA
LHGGNFQGTPIGVSMDNTRLAIAAIGKLMFAQFSELVN
DFYNNGLPSNLSGGRDPSLDYGFKGAEIAMAAYCSEL
QFLANPVTNHVQSAEQHNQDVNSLGLISSRKTAEAVD
ILKLMSSTYLVALCQAIDLRYLEENMKNTVKNTVSQVA
KKVLIMGLNGELHPSRFCEKDLLKVVDREHVFAYIDDP
CSATYPLMQKLRQVLVDHALINSEDSENLGTSIFLKIGS
FEEELKTLLPKEVEIVRSAFENENLQIPNRIRECRSYPLYK
FVRDDLGGEHLTGEKIRSPGEEFDKVFTAICEGKIIDPLL
QCLKDWDGAPLPIC

>gene-ENSIDEG00000008172 MAQATTVRSFLQVAATEEVASPLRVVQIEGLAILKIIKH
CKEFSPALVTGQLLGLDVGSVLEVTNCFPFPIREEDEEIE
AEGANYQLEMMRCLREVNVDNNTVGWYQSTLLGSY
QTVELIETFMNYQENIKRCVCIIYDPSKSNQGVLALKAL
KLSDSFMELYRSNNFTGEKLREKNLSWVDIFEEIPIKVSN
SALISAFMTELEADSPVTQCDYDRLQLSTNPFMERNVE
FLIECMDDLSMEQQKFQFYYRNMSRQQAQQQAWLQK
RRAENMARKAAGEEPLPEEDPSNPVFKPIQEPPRLDSF
LITNQISNYCNQINGVAGQSFSKLYLMKALYED

>gene-ENSIDEG00000008243 MDLLLLLEKGLVALFLAVIVAITISKLRGKRFKLPPGPLPV
PVFGNWLQVGDDLNHRNLTDYAKKFGDVFLLRMGQR
NLVVVSSPDHAKEVLHTQGVEFGSRTRNVVFDIFTGKG
QDMVFTVYGEHWRKMRRIMTVPFFTNKVVQQYRHG
WEDEVAHVVADVKANPEASTTGIVLRRRLQLMMYNN
MYRIMFDRRFESEEDPLFLKLKALNGERSRLAQSFEYNY
GDFIPILRPLLRGYLKICKEVKEKRLQLFKDYFVDERKKL
ESTKRMDNNSLKCAMDHILEAQQKGEINEDNVLYIVE
NINVAAIETTLWSIEWGIAELVNHPEIQQKLRHEIDTVL

	GPGVQVTEPDTHKLPYLQAVIKETLRLRMAIPLLVPHM<br>NLHDAKLGSYDIPAESKILVNAWWLANNPAHWKNPE<br>EFRPERFMEEESKVEASGNDFRYLPFGVGRRSCPGIILAL<br>PILGITLGGLVQNFELLPPPGQSKIDTAEKGGQFSLHILK<br>HSTIVAKPRSV
>gene-ENSIDEG00000009617	DKLIGTGEGITSLAVLATAFLVALALCTKIVGSSRREKNL<br>PPGPKPWPIIGNLNLIGALPHQSFHKLSQKYGHLMQLK<br>FGSRPVVVASSPEMAKQFLKTYDQIFASRPDFSAAGKYT<br>SYNHRGLLGAPYGPNWRNCRKICLSHIFSSKRLQSFEYI<br>RVEESRAFISRLYALSGKSVALKSHISRTTLSVISRIVLGKK<br>YFSVTKDSDNEIVTLEEFQGMVNDFFVLNGVLNIGDWI<br>TWIEFLDLQGNVKKLKAFGKKFDRFHDHVFYEHRAKR<br>ELDKDNFVEVKLDSDGVKSIIQDIIIGGTDTTAITIEWAM<br>SELMKQPHIIKKATEELDSVIGTGRWVEESDFPQLPYLD<br>AIVKETTRLHPAGPLLAPHFALEDCKVAGYDIRKGTTV<br>FVNVWSMGRDPTLWDEAEKFQPERFLGKEIDVKGQNF<br>ELLPFGSGRRMCPAYNLGIKMISLILANLLHGFNWKLP<br>VNMKIEELSMDEVYAQSGFRKYPLAALST
>gene-ENSIDEG00000009746	MISLTHQLYSKYPANMDTSTSLAVLATAFLVALALCTKII<br>GSSRREKNLPPGPKPWPIIGNLNLIGALPHQSFHKLSQK<br>YGHLMQLKFGSRPVVVASSPEMAKQFLKTYDQIFASRP<br>AYSAASKYTSYNHRGVLWAPYGPNWRNCRKICLSHIFS<br>SKRVESFEYIRVEESRAFISRLYALSGKSVALKSHISRTTLS<br>VISRIVLGKKCFSVTKDSDNEIVTLEEFQGMLNDFFVLN<br>GVLNIGDWITWIDFLDLQGNVKRLKAFGKKFDRFHDH<br>VFYEHRARRELDKENFVARDLVDHLLLQLDNDDSSSD<br>EEVKLDSDGVKGIIEDIIIGGTDTAAITIEWAMSELMKQP<br>HLIKKATEELDCVIGTGRWVEESDFPQLPYLDAIVKETT<br>RLHPAAPLLAPHFALEDCKVAGYDIRRGTTVFVNVWS<br>MGRDPTLWDEAEKFQPERFLGKEIDVKGQNFELLPFGS<br>GRRMCPAYNLGIKMISLILANLLHGFNWKLPDTMKIEE<br>LSMDEVYALIGFRKYPLAAVMEPRLPLHLYWLST
>gene-ENSIDEG00000011027	MEKLGFFVCVWFLLYGSCLARFVVEKNSLKVTSPDNLK<br>DTYECAIGNFGVPQYGGTLVGAVIYPKANQKACKSFTD<br>VDISFKSKPGGLPIFLLADRGDCYFTLKAWNAQMAGA<br>AAILVADDRVEPLITMDTPEEEDARADYLQNITIPSALIG<br>KSLGDNIKKALSSGDMVNINLDWRESLPHPDERVEYEF<br>WTNSNDECGPKCESQIEFVKNFKGVAQILERKGYTQFT<br>PHYITWYCPEAFILSKQCKSQCINHGRYCAPDPDQDFS<br>KGYDGKDVVFQNLHQVCFYKVANESGKPWLWWDYV<br>TDFAIRCPMKDKKYTKECADQVIQSLGVDLKQIDKCV<br>GDPNADIDNPILKIEQEAQIGKGPRGDVTILPTLVINNR<br>QYRGKLDKGAILKAICSGFEETTEPSICLSEGIQTNECLE<br>NNGGCWQDKAANITACKDTFRGKVCECPTVQGVKFS

GDGYTHCEASGALRCEINNGGCWRKTQNGMTYSACI
DDHTKGCKCPPGFKGDGVNSCKDINECKEKSACQCSE
CKCKNTWGSYECSCSGKLLYMREHDTCISKDVNAEVS
WGFVWVIILGLAAAGVGGYAVYKYRIRRYMDSEIRAIM
AQYMPLDNQPEVPNHLSQGSV

>gene-ENSIDEG00000014290 MGCQRSRKTLFLGVMLLNLYQSVMGRFVVEKNSFRVT
SPDSLKGTHDSAIGNFGIPQYGGSMAGTVVYPKDNRK
GCKSFDDFGISFKSKPGALPTFVLVDRGDCFFALKVWN
VQNAGASAVLVADDVEEALITMDTPEADIKSTKYIQNI
TIPSALLEKTFGEKLKKALGNGDMVNVNLDWRESVPH
PDDRVEYELWTNSNDECGIKCDMLMEFVKDFKGAAQI
LEKGGYTQFTPHYITWYCPMAFTVSKQCKSQCINQGR
YCAPDPEQDFSSGYEGKDVVLENLRQLCVFKVASESQK
PWVWWDYVTDFQIRCPMREKKYKKECADSVIRSLGLD
SKKIEKCMGDPNADSDNPVLKEEQDAQVGKGSRGDV
TILPTLVVNNRQYRGKLAKGAVLKAICSGFEETTEPSVC
LSGDVETNECVDKNGGCWQDKSGNITACKDTFRGRV
CECPLVDGVQFKGDGYSSCVASGPGRCKVNNGGCWH
ETRDGHTFSACLDNGDGKCTCPPGFKGDGVKSCEDID
ECKEKKVCQCSECSCKDTWGSYECTCSGDLLYIREHDI
CISKTATEVKSAWAAVWVILIGLAMAGGGAYLVYKYRL
RSYMDSEIRAIMAQYMPLESQSEVPNHVSDDRA

>gene-ENSIDEG00000017095 QMLNWSGCVENGHQNGTAMELCLQKDPLNWGVAA
EALKGSHLDEVKRMVAEFRKPVVRLGGESLTISQVAAIS
ARDNNGVKVELSEASRAGVKASSDWVMESMNKGTDS
YGVTTGFGATSHRRTKQGGALQKELIRFLNAGIFGNGK
ETAHTLPHSATRAAMLVRINTLLQGYSGIRFEILEAITKF
LNHNITPCLPLRGTITASGDLVPLSYIAGLLTGRPNSKAV
GPTGETLSPKEAFSLAGVDGGFFELQPKEGLALVNGTA
VGSGMASMVLFEANILALLSEVLSAIFAEVMQGKPEFT
DHLTHKLKHHPGQIEAAAIMEHILDGSSYVNAAQKLH
EMDPLQKPKQDRYALRTSPQWLGPQIEVIRSSTKMIERE
INSVNDNPLIDVSRNKALHGGNFQGTPIGVSMDNTRL
AIAAIGKLMFAQFSELVNDFYNNGLPSNLSGGRNPSLD
YGFKGAEIAMASYCSELQFLGNPVTNHVQSAEQHNQ
DVNSLGLISSRKTAEAVEILKLMSTTFLVGLCQAIDLRHL
EENLKLTVKNTVSQVAKRVLTMGVDGELHPSRFCEKD
LLRVVDREYIFAYIDDPCSATYPLMQKLREVLVEHALKN
GDNEKNSSTSIFQKIATFEDELKTILPKEVESARAVLERG
NPAIPNRIKECRSYPLYKFVREELGAVYLTGEKDRSPGEE
FDKVFTAMCKGEIIDPLLECLQGWNGAPLPIS

>gene-ENSIDEG00000017846 MKGKKHFLLLSLPVQSHINPTIQLAKILTRSGANVTYAT
TTTGLGRLNALPTIEGLSYATFSDGNEDNATLIHNDYM
AKLRCVGRRSITKLLQDLSTKGSPVTFIVYTVLLPWVAE

VARGMDLPSAFLFIQCAAAFAIFHHLFNTQDGLLDGV
HDINPDVSIKLPGLPLLTSHDLPTFLFPHNEYYSFTAPLF
EEHIKTLEKDPNPCVLVNTFDALEGDIIKSFPNMKFMAI
GPLLPSAFSDGNDLHDKSFGGTLFQNPNNYLTWLDSK
PDRSVIYASFGSLMQLQETQKEEILHGLMASNRPFLWV
CRDINEEEVNSMKLKNEISDELGLIVTWCSQVEVLCHR
SIGCFVTHCGWNSTVESIIGGVPVVGCPHFSEQKTNIKI
VEEVWGNGVRVKENDEGLFGRKEIKRCLDIVMGEGEK
GKEIRGNAMKWKSLAVEAVKKGGSSHNNVKQILEM

>gene-ENSIDEG00000019209 MHEHCHVTFPAQGHMNPTMQFAKKLVQMGVEVTFAI
SVFAHRRMAEACGGDTKGLNFALFSDGYDDGFKITDD
AKHYRSQIKSRGTECLKEIIRSSAEEGRPINCLVYTLLLP
WAAEVARDSHIPSALLCIQPATVLDIYYYYFNGYNEALS
NCNDPSWSIKFPGLPLLYAKDLPSFILPSSQEAYNFALA
AFKEQLEALDSDTHPRVLVNSFDALEPDALKAIDKLKLI
AIGPVIPSAFLDAKDPSDTSFGCDLFQKLRNYSDWLNS
KPKESVIYVSFGSILELTKPQMEEIALGLLKSKRPFLWVIR
KKEEEKDQELRCMEQLEQLGMIVPWCSQLEVLSHAAL
GCFVTHCGWNSTLESLASGVPVVAFPKWTDQGTNAK
LIEDVWGTGVRVKGNEEGFVDGDEISRCLEMVMEGEE
YRRNAKKWRDLAREAMKEGGSSDKNLKSGSKFDLFLS
CFLIALMLTSCMLTLYVSNIFLA

>gene-ENSIDEG00000020061 MNKLIMEDSSCGGGERKVTTKTNKRCCCYCVTGATGY
IGSWLVKSLLDRGYWVHATLRHPAKSLHLSKLWGAND
RLRIFRADLLEEGSFDEAVKGCIGVFHVAASMEFSVLEN
ENVDDYVQSNIIDPAIKGTLNLLKACLKSKSVKRVVFTS
SISTLTAKDSAGNWRDVVDEFCQTPIDHVWNTKASGW
VYVLSKLLTENAAFQFAYDEGIDLVSLITTTVAGPFLTSTI
PSSIRVILSPLTGDPEFFSILSAVNSRMGSIALVHIEDICSA
HIFLMEHSKAQGRYICCTQSCEMSDMFHRLAKVYPCP
NIQSWLMMEDQQGPTPSEISSKKLRDLGFTFKFGLQDII
HHTVNSCVYNEFLPPIRK

>gene-ENSIDEG00000020781 MVIEKALELPIIDLSSSDRIATAQSIHQACMDCGFFYLIN
HGVENELLQKVFEESRKFFSLPLEEKLKLPRKEHRGYTP
LYAEKLDPSSSSKGDSKETFYIGPLEDDKSHLNQWPSSE
VLPSWRFIMELYYEKIRDAGRRLLALIALALNIEEGFFEK
GGPSESFLRLLHYPGELCFLEEQIYGASAHSDYGMITLL
ATDGVGGLQVCREKFKQPQVWEDVHHISGAFVINIGD
MMERWTNCLYRSTLHRVIRTGQERYSVINFCLPIYNSYF
FCHVFCLSVHGIHMLVRHIHKKRRSQPYTRSVPKWLSF
WIPIQNVW

>gene-ENSIDEG00000020902 MGGQRSRNTVFLGFMLLNLYQSVIGKFVVEKNSFRVTS
PDILKGTHDCAIGNFGIPQYGGSMAGTVVYPKDNRKG
CKSFDDFGISFKSKPGALPTFVLVDRGDCFFALKVWNV

	QNAGASAVLVADDVEEALITMNTPEEDIQLAKYIQNITI
	PSALLEKTFGEKLKQSLSNGEMVNVNLDWRESVPHPD
	DRVEYELWTNSNDECGIKCDTLMEFVKDFKGAAQILE
	KGGYTQFTPHYITWYCPMAFTVSKQCKSQCINHGRYC
	APDPEQVFGSGYDGKDVVLENLRQLCVFKVANENQKP
	WVWWDYVTDFQIRCPMREKKYNKDCADSVIRSLGLD
	SKKIEKCMGDPNADSDNPVLKEEQDAQVGKGSRGDV
	AILPTLVVNNRQYRGKLVKGAVLKAICSGFEETTEPSVC
	LSGDVETNECIVKNGGCWQDKSANVTACKDTFRGRV
	CECPLVDGVQFKGDGYSSCVASGPGRCKVNNGGCWH
	ETRDGHTFSACLDNGDGICTCPPGFKGDGVKSCEDIDE
	CKEKKVCQCPKCSCKDTWGSYECTCSGDLLYIREHDTC
	ISKTATEVKSAWAGVWVILIGLAMAGGGAYLVYKYRLR
	SYMDSEIRAIMAQYMPLDSQSEVPNHVSDDRA
>gene-ENSIDEG00000020904	LISNKPPARLLHPTYLPKKHHKFYLLVLHIQIEMECVGQ
	QSGTVSSDLCLQKDPLNWGVAAEALNGSHLDEVKRM
	VAEFRKPVVRLGGESLTISQVAAISVRDNSGVKVELSEG
	ARAGVKASSDWVMESMNKGTDSYGVTTGFGATSHRR
	TKQGGALQKELIRFLNAGIFGNGTETAHTLPHSATRAA
	MLVRINTLLQGYSGIRFEILEAITKFLNHNITPCLPLRGTI
	TASGDLVPLSYIAGLLTGRLNSKAVGPTGETLNPEEAFR
	LAGVEGGFFELQPKEGLALVNGTAVGSGMASMVLFEA
	NILALLSEVLSAIFAEVMQGKPEFTDHLTHKLKHHPGQ
	IEAAAIMEHILDGSSYVKAAQKIHEMDPLQKPKQDRYA
	LRTSPQWLGPQIEVIRSSTKMIEREINSVNDNPLIDVAR
	NKALHGGNFQGTPIGVSMDNTRLAIAAIGKLMFAQFS
	ELVNDFYNNGLPSNLSGGRNPSLDYGFKGAEIAMASY
	CSELQFLGNPVTNHVQSAEQHNQDVNSLGLISSRKTAE
	AVEILKLMSTTFLVGLCQAIDLRHLEENLKLTVKNTVSQ
	VAKRVLTMGVNGELHPSRFCEKDLLRVVDREYIFAYID
	DPCSATYPLMEKLRQVLVDHALTNGDNEKNLSTSIFQK
	IAAFEDELKTILPKDVENARAALESGNPAIPNRIKECRS
	YPLYKFVREELGTVYLTGENVRSPGEEFDKVFTAMSKGE
	IIDPLLECLKSWNGAPLPIC
>gene-ENSIDEG00000021031	MSWSTTINLLELVPNFNLIYYGFRNFFLIQKIVILIHFINK
	ILLSHQNLLIPYLLLKSYVILKFYGPKCIQTLNSISIRILAP
	HQNLPVPTLLYIHLHSHRILAPHQNLSVPTLLYIHIVFSR
	RIKIYPYPLCYIFTYIHKFSPIQTKKKIKSKAMALATTLAL
	ILYSTITACFIYLIINLRTLLLNRHRRPLPPGPKPWPLIGN
	LPHLGTMPHHSIAAMARTYGPLMHLRMGFIHVVVAA
	SAEVAAHFLKTHDANFSSRPPNSGAKYMAYNYQDLVF
	APYGSRWRMLRKICAVHLFSAKALDDFRHVREEEVAIL
	TRALVDSGQKALQLGQLLNVCTTNALGRVMLGRRVFR
	DGSTNVDPKADEFKQMVVELMVLAGEFNIGDFIPALE

WLDLQGVTAKMKKLHARFDSFLSLILEEHKSKANGDG
QQNHMDLLSMLLSLKDDADQSEGGKLTNTEIKALLLN
LFTAGTDTSSSTVEWAIAELIRNPKIFAQAQKELDSVVG
QDRLVTDSDLSQLPYLQAIVKETFRLHPSTPLSLPRIAAQ
SCEINGYYIPKGSTLLVNVWAIARDPNVWAEPLEFRPER
FLPGGERPSADVKGNDFEVIPFGAGRRICAGMSLGLRM
VQLVTATLVHAFNWELPEGESAEKLNMDEAFGLTLQR
AAPLVVHPQPRLAHQSYLQMGVVN

>gene-ENSIDEG00000021815

MENSTLTGLLKQVAAEFPKRRALSVSGQFDLTHARLQ
DLVEQAASRLLAAGVEPGEVVALTFPNTVEFIIMFLAVI
RVRATAAPLNAAYTSDEFEFYLSDSESKHLLTSKEGNEP
AQAAASKLNIPHVTATLPRADSEVSLSSTQSESDANSVS
NIINDPSDVALFLHTSGTTSRPKGVPLTQLNLASSVQNI
KSVYKLTESDSTVIVLPLFHVHGLIAGLLSSFGAGASVTL
PAAGRFSASTFWSDMNKYNATWYTAVPTIHQIILDRHL
SKPELTYPKLRFIRSCSASLAPSILARLEEAFGAPVLEAYA
MTEAAHLMASNPLPENGPHVPGSVGKAVGQEMAILD
ENGVQQEAGASGEVCIRGPNVTKGYKNNPEANKTGF
QFGWFHTGDIGYLNSDGYVHLVGRIKELINRGGEKISPI
EVDAVLLSHTDIAQAVCFGVPDDKYGEEINCAVIPREGS
DIDEAEVLRFCKKNLAAFKVPKKVFITDAVPKTATGKIQ
RRIVAEHFIAQISTAKVPKFGA

>gene-ENSIDEG00000022689

MMREKLGFLVCVWILLYGSCMGRFVVEKNSLKVTSPD
KLKDKYECAIGNFGIPQYGGTLVGAVIYPKANQKACKS
FSDVDISFKSKPGGLPIFVLADRGDCYFTLKAWNAQMA
GAAAILVADDRVEPLITMDTPEEDDARADYLQNITIPSA
LISKSLGDNIKKALSNGDMVNINLDWRESLPHPDERVE
FEFWTNSNDECGAKCESQIDFVKNFKGVAQILEQKGYT
QFTPHYITWYCPEAFILSKQCKSQCINHGRYCAPDPEQ
DFSKGYDGKDVVIQNLRQACFYKVANESGKPWLWWD
YVTDFAIRCPMKDKKYTKECADQVIVSLGVDLKQIDKC
IGDSNADVDNPILKTEQEAQIGKGSRGDVTILPTLVINN
RQYRGKLDKGAVLKAICSGFEETTEPAICLSEGIETNECL
ENNGGCWQDKAANITACKDTFRGRVCECPIVQGVKF
AGDGYTHCEASGALRCEINNGGCWRKTQNGRTYSAC
VDDHTKGCKCPPGFKGDGVNSCEDVDECKEKSACQC
PGCKCKNTWGSYDCSCNGNLLYMREHDTCISKDVNTE
VSWGFVWVIILGLAAAGVGGYAVYKYRIRRYMDSEIRA
IMAQYMPLDNQPEVPSHVSHGNV

>gene-ENSIDEG00000023459

FVSHKMEKSGYCQDGIYRSLRPPLSLPRDSNLSMISFLFR
NSSSFPNKSALIDADSGETLTFSHLKSTVFKVSNGLSQL
GIQKNDVVLIFAPNSIQFPLCFFGIIAAGAIATTVNPSYT
VPEISKQIKDCNPKIIITVPELWGKVKGFNLPAVVLGSEK
SFEGFESNSRITLFTDLVKNNGSVSNLAKISSNDTAALLY

	SSGTTGNSKGVILSHRNFIAAALMLSSDQDLMSEVDSV<br>FLCVVPMFHVFGLALITFGQLQRGNAIVSMAKFDFEMI<br>LSSVEKYKVTHLWVVPPIILALAKQSLVKKYDLSSLKQI<br>GSGAAPLGRELMQECSKNFPQALIIQGYGMTETCGVIS<br>VENQLGGPRHSGSAGMLVPGVESQIVSVDTMRPLPPK<br>VLGEIWVRGPNMMQGYFNNPQATKLTIDKQGWVHT<br>GDLGYFDEEGQLFVVDRIKELIKYKGFQVAPAELEGLLV<br>SHPEILDAVVIPFPDAEAGEVPIAYVVRSPDSSLSEEDVQ<br>KFIAEQVAPFKRLRRVTFVNSVPKSASGKILRRELIEKVR<br>AKL
>gene-ENSIDEG00000028706	PPTKKQKLLYAYSVSPLLYKPLVLCVENGHQNGTATELC<br>LQKDPLSWGVAAEALKGSHLDEVKRMVAEFRKPVVRL<br>GGETLTISQVAAISARDNSGVKVELSEAARAGVKASSD<br>WVMESMNKGTDSYGVTTGFGATSHRRTKQGGALQKE<br>LIRFLNAGIFGNGTETAHTLPHSATRAAMLVRINTLLQ<br>GYSGIRFEILEAITKFLNHNVTPCLPLRGTITASGDLVPLS<br>YIAGLLTGRPNSKAVGPTGVTLSPEEAFSLAGVQGGFFE<br>LQPKEGLALVNGTAVGSGMASMVLFEANILALLSEVLS<br>AIFAEVMQGKPEFTDHLTHKLKHHPGQIEAAAIMEHIL<br>DGSSYVKAAQTLHEMDPLQKPKQDRYALRTSPQWLGP<br>QIEVIRSSTKMIEREINSVNDNPLIDVSRNKALHGGNFQ<br>GTPIGVSMDNTRLAIAAIGKLMFAQFSELVNDFYNNGL<br>PSNLSGGRNPSLDYGFKGAEIAMASYCSELQFLGNPVT<br>NHVQSAEQHNQDVNSLGLISSRKTAEAVEILKLMSTTF<br>LVGLCQAIDLRHLEENLKLTVKNTVSQVAKRVLTMGV<br>DGELHPSRFCEKDLLRVVDREYIFAYIDDPCSAAYPLMQ<br>KLRQVLVEHALKNGDSEKSSSTSIFQKIATFEDELKTLLP<br>KEVESARAMLESGNPVIPNRIKECRSYPLYKFVREELGT<br>LYLTGEKVRSPGEEFDKVFIAMSKGEIIDPLLECLEGWN<br>GAPLPIS
>gene-ENSIDEG00000032074	MECVGHQNGTASDLCLQKDPLNWGVAAEAVKGSHL<br>DEVKRMVAEFRKPVVRLGGESLTISQVAAISVRDNGGV<br>KVELSEGARAGVKASSDWVMESMNKGTDSYGVTTGF<br>GATSHRRTKQGGALQKELIRFLNAGIFGNGTETTHTLP<br>HSATRAAMLVRINTLLQGYSGIRFEILEAITKFLNHNITP<br>CLPLRGTITASGDLVPLSYIAGLLTGRPNSKAVGPTGETL<br>NPEEAFRLAGVEGGFFELQPKEGLALVNGTAVGSGMA<br>SMVLFEANILALLSEVLSAIFAEVMQGKPEFTDHLTHKL<br>KHHPGQIEAAAIMEHILDGSSYVKAAQKLHEMDPLQK<br>PKQDRYALRTSPQWLGPQIEVIRSSTKMIEREINSVNDN<br>PLIDVARNKALHGGNFQGTPIGVSMDNTRLAIAAIGKL<br>MFAQFSELVNDFYNNGLPSNLSGGRNPSLDYGFKGAEI<br>AMASYCSELQFLGNPVTNHVQSAEQHNQDVNSLGLIS<br>SRKTAEAVEILKLMSTTFLVGLCQAIDLRHLEENLKLTV

KNTVSQVAKRVLTMGVNGELHPSRFCEKDLLRVVDRE
YIFAYIDDPCSATYPLMQKLRQVLVDHALKNGDNEKN
LSTSIFQKIAAFEDELKTLLPKEVENARAVLESGNPAIPN
RIKECRSYPLYKFVREELGAVYLTGEKVRSPGEEFDKVFT
AMSKGEIIDPLLECLNSWNGAPLPIFFSLCFYCSENINITI
NTTCIIGKLLLKKLFKINRHLT

>gene-ENSIDEG00000032275
MAATISVEEIRKSQRAEGPATILSIGTATPSNCLYQADYP
DYYFRITNSEHKTDLKNKFKRMCEKSMIRKRYMHLTEG
ILKENPNLCAYEAPSLDARQDMVVLEVPKLGKEAATK
AIKEWGQSKSKITHLIFCTTSGVDMPGADYQLTKLLGL
RPSVKRFMMYQQGCFAGGTVLRLAKDLAENNAGARV
LVVCSEITAVTFRGPSESHLDSLVGQALFGDGAAAVIVG
SDPDTCVERPLFQLVSAAQTILPDSDGAIDGHLREVGLT
FHLLKDVPGLISKNVEKSLKEAFGPIGISDWNSLFWIAH
PGGPAILDQVEIKLGLKEEKMRATRHVLSEYGNMSSAC
VLFILDEMRKKSTEERKATTGDGLDWGVLFGFGPGLTV
ETVVLHSVPATIT

>gene-ENSIDEG00000033075
MENSTLTGLLKQVAAKFPNRRALSVSGQIDLTHARLQ
DLVERAASRLVAAGVKPGEVVALTFPNTVEFIIMFLAVI
RVRATAAPLNAAYTSEEFEFYLSDSESKLLLTSKEGNEPA
QAAASKLNIPHFTATLPQADSEVSLSSTQSESDPNSVSNI
VNGPTDLALFLHTSGTTSRPKGVPLSQLNLASSVQNIKS
VYKLSESDSTVIVLPLFHVHGLLAGLLSSFGAGASVTLP
ATGRFSASTFWSDMNKYNATWYTAVPTIHQIILDRHLS
KPEPTYPKLRFIRSCSASLAPSILARLEETFGAPVLEAYA
MTEATHMMASNPLPENGPHVPGSVGKPVGQEIAILDE
NGVELEAGASGEVCIRGPNVTKGYKNNPEANKTAFQF
GWFHTGDIGYLNSDGYLHLVGRIKELINRGGEKISPIEV
DAVLLSHTDIAQAVCFGVPDDKYGEEINCAVIPREGLDI
DEAEVLRFCRKNIAAFKVPKKVFITDAVPKTATGKIQRR
IVAEHFLAQISAAKVPKFGA

>gene-ENSIDEG00000036279
MIMDPNSGYSAETKTFHSLRPPVPLPPETTPLSAASYSLP
LQLTSPWPDSTALIDSATGERISYSEFTRLTKNLASSLQN
LIGLSKGQTAFILSPNSTRIPILYFSLLSLGVIISPSNPISVES
EISRQIKLSQPVIAFATAATCHKITNLRYRTILIDSPEFES
MMKSTPSRELEHVEVNQSDVAAVMYSSGTTGLVKGVM
LTHGNLIGIVGNYYYQRSERESPAIVLYTAPFFHVIGFYY
CVKSVALNETAVVMSRFDLRKMCRAMEEFRVTDVVTA
PPVVVAMIKEDLTNDFDLKSLEAVGSGGAPLGKDVIGA
FNAKFPGVILFQGYGMTETTGAAFRAIGLDESLHWGSV
GKLAGNCEARIIDEDTGNSLPPGKQGELWIRGPTVMK
GEVRISYFSMHNETKRGEMTTNATSETLVADGWLRTG
DLCYFDEEGFLFVVDRLKELIKYKGYQVPPAELEQLLQT
HPEVVDAAVIPYPDEEAGEVPMACVVKRPQSSIDEAH

	VMDFIAKQVAPYKKIRRVMFVSSIPKSAAGKILRKELKK
	TVGFQGGSLSKL
>gene-ENSIDEG00000036664	MASLQYFPTPTIPSPTLWSGSFQSKTSQFFINQKNEQNST
	LNYSLSKLDRRNVLLGLGGLYGSATAANLSPDALAAPV
	EVTKCGEATTLPKGVAKIDCCPPPETIIDFKLPPPPKILRV
	RPAAHLADEKYIAKYSEALARMKALPKDDPRSFAQQA
	AIHCAYCDSSYQMVGFPDVKVDVHFSWMFFPFHRLYL
	YFFEKILGKLIDDPTFALPYWNWDSPPGMPIPTMFTNP
	NSPLYDKLRNANHQPPLKVDLNYSAKVPSPTDEKKLIA
	SNLSIMYKQMVSGAKNPKLFFGNPYRAGDPQNQGMG
	TIENLPHTPIHIWTGDPTQPNGEDMGRFYSAGRDPIFYS
	HHANVDRMWNLWKLLPGRNRKDIRDPDYLNTAFIFY
	DENKRLVRATVKDCLDTKKLGYVYQDVDIPWLGYRPK
	AKRGKKGKSGIANADELPIEYPKLLDSLVKVRVPRPKKS
	RSTEEKEDEEEILVIDGIEYDGNVYVKFDVYINDEDEVES
	GPDNAEFAGSFTNVPLKENRKVKTSLNLGISELLEDLG
	AEDDDGVVVAIVPRSGKGEVSIGGIKIEISS
>gene-ENSIDEG00000038802	MGYSKEDNIEVLSASASAKVSSLNCIDLSCSDIQHSVSL
	LKQACLDCGFFYVINHGISQEFMDEVFAQSKRFFDLPSE
	QKMKVLRNEKHYRGYTPVLDEYLDPVNQINGDYKEG
	YYIGIEVPEDDPKAQKAFHGPNVWPATDILPGWRETM
	EKYHQEALEVAKAVARIISLALDLGSDFFDQPEMLSDPI
	AVLRLLHYEGQVSEPTKGIYGAGAHTDFGLITLLTTDD
	VRGLQICKDKDAKPQVWEYVAPLKGAFIVNLGDMLER
	WSNNRFRSTLHRVLVNGQERYSIAFFVEPSHDCIVECLP
	TCQSKENPPKFPPIKCETHLLQRYRETHADLNSYK
>gene-ENSIDEG00000039930	ASRLAMTSLRTTNNAFFSSTFNTPCTFSSWCTTRKPSQS
	LTKRTQNPSFKISSKAPADDKNGELSQNCDTRRNVLLG
	LGGLYGVATNLTANPFAFAAPVSAPDLTKCGPADIPDG
	STKIDCCPPESAQIIDFKFPPIPKHLRIRPAAHLADEEYV
	AKFSKAIELMKALPEDDPRNFLQQAYIHCAYCNGAYE
	QVGFPNLDLQVHNSWVFYPFHRWYLYFYEKILGKLID
	DPTFALPFWNWDNPGGMQMPAMFLDPNSSLYDKYRN
	AGHYPPTILDLGYDGKDKDTSDSEKITNNLAIMYREMV
	ANGSTALLFFGSPYRAGDKPDPGPGSIEKVPHIPIHRWT
	GDPTQPNGEDLGNLYSSARDTLFYSHHANVDRMWTI
	WKTLGGKRKDIKDLDWLNTSFLFYDENAQLIRVKVQD
	CLDPKKLGYSYQNVGIPWLKSRPIPRKKKSKVSSKAQE
	TKVGSVTFPLTLDKIVKILVTRPKKSRSQKQKNDEDEILV
	IDGIEFDSQKYVKFDVFINDEDDKGSAAADKSEFAGSFA
	QLPHKHKHDKKLKTTLRLGITELLEDLEAEDDDNLLVT
	LVPKSGIEDVTISGIKIDHYGISC
>gene-ENSIDEG00000040116	MVYSKKENIEAISASASAKDSSLNSIDLSCPDIQHSVSLL
	KQACLDCGFFYVINHGISQEFMDEVFAQSKRFFDLPIEQ

	KMKLLRNKKNRGYTPVLDEYLDPVNQINGDYKEGYYI<br>GIEVPEDDPKAQKRYHGPNVWPSTDILPGWRETMEKY<br>HQEALEVAKAVARIISLALDLGSDFFDQPEMLGDPIAM<br>LRLIHYEGRVSEPTKGIYGAGAHTDFGLITLLTTDDVPG<br>LQICKDKDAKPQVWEHVAPLKGAFIVNLGDMLERWS<br>NNRFRSTLHRVLANGQERYSIALFVKPSHDCVIECLPTC<br>QSKENPPKFPPIKCETHLLQRYTETHADLNKYK
>gene-ENSIDEG00000005018	MADQEVKLIGVWGSPFSRRVEIALKMKGIKYEFLEEDL<br>SNKSPLLLKYNPVHKKVPVLLH<br>NGNPIAESLVIVEYIDETWKSGPPILPTDPYGRAMARFW<br>AKFIDDKCLPAIWKIRRSQKD<br>ERKQATEEAFELLKILENELNGKKLFGGENIGFVDIAA<br>NFIGLWLGILQEMLGIELLTKE<br>KLPKLCEWVDEYLNSDIIRQSLPPRDELAARLRALFQPK
>gene-ENSIDEG00000010192	MSDHEGVKLFGSWSSPYSRRVEIALKMKGVEYEYIEED<br>LSNKSPLFLKYNPVHKKVPVLL<br>HNGKPIAESLVILEYIDETWKSGPPILPKDPYQRARARF<br>WAKFIDDKFLVALLKIRASKT<br>LEEAEQAIEEASNLLKTLENELKDNKFFGGESINLTDIA<br>AAYVALWLGVLQEITGVQNLY<br>TKEKFPRLSKWNKEFLNCSIIKEKLPPRAELLAHLRPRF<br>QKS
>gene-ENSIDEG00000007830	MPESGVKVLGTWYSPFVLRVQIALNAKSIDYEYVEETLI<br>SSKSDLLLKYNPVHKKVPVLI<br>HADKSICESLIIVQYIDEAWTNGPSILPSDPYDRAIARFW<br>AAYIDDKLFPVVTELRSAQD<br>EEEKTALKERVIEGMVLLEEAFVKCSKGKGYFGGDSIGY<br>LDIALGSSLGWLKAMETVSGI<br>KLVDETKTPALFGWAQRFSLTDAVKDVMPETGKLVELL<br>KMIQARAKASSN
>gene-ENSIDEG00000018918	MADGVVLLDCWASMFGMRVRIALAEKGIEYEYKEEDL<br>RNKSPLLLKMNPVHKKVPVLIHN<br>GKSICESSIAVQYIDEVWKEKAPLMPSIPYQKAQARFWA<br>DFVDKKLYDAGRKTWVTNGEQ<br>VAAKKEFIEYLKVLEGELGDKSYFGGDTFGYVDVILVPF<br>YSWFCVYETFGNFSIEAECPK<br>LIAWVKRCLEKESVSKSLPDSQKVYEFVLGLKKKLGY
>gene-ENSIDEG00000033926	MADGVVLLDCWVSMFGMRVRIALAEKGIEYEYKEEVL<br>RNKSPLLLKMNPVHKKVPVLIHN<br>GKSICESSIAVQYIDEVWKDKAPLMPSDPCKKAQARFW<br>ADFVDKKIYDAGRKTWATKGEE<br>QVAAKKEFIEYLKVLEGELGDKSYFGGDTFGYVDVILVP<br>FYSWFYAYETIGNFSIEAECP<br>KLIAWVKRCLEKESVSKSLPDSRKVYEFVLGMRKKLGY

Table S4 The FPKM value of HhGRFs and enzymes related to flavonoid biosynthesis through RNA-seq technology under MeJA treatment.

ID	FPKM-M0	FPKM-M6	FPKM-M12
HhGRF1	1.7084	2.1260	3.2238
HhGRF2	2.0052	1.6409	1.9032
HhGRF3	3.1323	4.3166	2.0695
HhGRF4	2.1886	4.4206	2.0764
HhGRF5	2.5267	5.3029	5.4559
HhGRF6	0.3319	0.5091	0.7870
HhGRF7	1.1435	1.4714	1.5592
HhGRF8	0.1653	0.1349	0.0964
HhGRF9	3.6446	3.2678	3.6213
HhGRF10	8.2514	9.9466	9.6898
HhGRF11	0.4170	0.1959	0.2682
HhGRF12	11.6950	7.5734	6.6886
HhGRF13	3.4545	2.6102	3.2246
HhGRF14	0.1737	0.1600	0.2400
HhGRF15	25.3471	14.2908	11.0710
HhGRF16	0.5379	0.4310	0.9219
HhGRF17	0.9870	1.3953	1.5882
HhGRF18	0.4460	0.1652	0.2237
HhGRF19	0.1804	0.1628	0.1626
HhGRF20	0.5077	0.2147	0.4890
gene-ENSIDEG00000000387	13.4092	18.8801	23.8323
gene-ENSIDEG00000002867	12.3674	11.8103	10.2796
gene-ENSIDEG00000003654	11.8099	10.0331	16.0560
gene-ENSIDEG00000003808	3.1358	5.6184	4.1624
gene-ENSIDEG00000004662	0.2040	0.5320	0.6951
gene-ENSIDEG00000005555	15.7238	14.3189	16.5939
gene-ENSIDEG00000005968	2.0221	4.1493	2.7608
gene-ENSIDEG00000008172	28.5785	24.4137	35.0642
gene-ENSIDEG00000008243	21.9632	20.0598	16.5388
gene-ENSIDEG00000009617	29.3177	27.0222	0.6815
gene-ENSIDEG00000009746	4.0837	2.9787	0.1086
gene-ENSIDEG00000011027	45.5245	51.6010	45.9229
gene-ENSIDEG00000014290	6.7779	7.7637	9.6874
gene-ENSIDEG00000017095	2.3198	3.8809	2.0133
gene-ENSIDEG00000017846	10.8491	16.6915	12.5781
gene-ENSIDEG00000019209	7.3069	9.6328	5.6997
gene-ENSIDEG00000020061	26.6545	17.3365	15.0521
gene-ENSIDEG00000020781	31.3123	27.4301	31.0022
gene-ENSIDEG00000020902	10.5768	21.4142	20.7525
gene-ENSIDEG00000020904	2.2207	2.5109	1.4897

gene-ENSIDEG00000021031	0.2358	0.3756	0.3697
gene-ENSIDEG00000021815	33.0163	22.6820	21.0944
gene-ENSIDEG00000022689	11.5825	17.1343	19.2971
gene-ENSIDEG00000023459	4.5660	9.3846	9.5377
gene-ENSIDEG00000028706	2.7801	5.9062	3.8634
gene-ENSIDEG00000032074	3.5921	6.9724	7.0026
gene-ENSIDEG00000032275	1.7198	0.8307	0.3386
gene-ENSIDEG00000033075	2.6677	1.9820	1.8993
gene-ENSIDEG00000036279	52.0139	31.0247	24.8070
gene-ENSIDEG00000036664	17.4659	229.2105	59.3104
gene-ENSIDEG00000038802	39.7296	42.2259	43.2992
gene-ENSIDEG00000039930	0.3411	1.2499	2.1753
gene-ENSIDEG00000040116	8.9908	10.2594	11.3275
gene-ENSIDEG00000005018	50.5333	57.1964	49.1557
gene-ENSIDEG00000010192	3.4811	5.7489	7.6371
gene-ENSIDEG00000007830	1.0151	2.5634	3.1893
gene-ENSIDEG00000018918	128.2793	206.2138	182.8510
gene-ENSIDEG00000033926	30.4632	55.1166	45.6028

Table S5 The protein sequences of GRF family in *H. helix*, *A. thalian*, and *O. sativa.*

Name	Protein sequences
>HhGRF1	MDFGGNVVGLVSSSSGTTTSTVFASDLEAKQKWYGSGLLKQERSVPISTEP DDCIRDFKVAKNTDDFSLSKAMLLHQQQQQKNLSLQRSNSSSLFSDGQQ QMLSFSPPNSQPVTLPYYHHSPTPYSRNTGYGSGGLNAANMHGIITGVRG PFTPSQWMELEHQALIYKYITANSPVPSNLLNPIRKALESAGFSSFPGAHL RPNTLGWGAFHLGFSNNTDPEPGRCRRTDGKKWRCSRDAVADQKYCER HVNRGRHRSRKPVEGQTGHSVSGTTNTTAKFLPMSSSTSASSVVPASGTS NSLGLSNHQLNNLQHSAPNPSASAHINSRSFLNKANVGENIQDTTGLSM LSPTIGLKENQFSTQKQHNPYQESPRTEFGLVCSDSFLNPFQKSSSLINCRS YGSSENLYDGGNKSQHSLRQFMDDWPKNQSERSAISWPDTDMQSDRTQ LSISIPMAASDFMSSTSSPTNEKFALSLLRSSRELDSTQMGLGMNGIINETN QRHASWIPISWENSIGGPLGEVLHSTNNSAGDCNNTSGLNLMTEGWDSS PRLASSPTGVLQKTTTFRSLSNSSAGSSPRTENNLSTNLISSSLPAL
>HhGRF2	MIRSHHNDNGANEVLLRCRSNIYRQDAAAAGPPNVGAVVVRAIQHPFD TSTTTNTTTNNTSSKSPGGMDSALGYAAAFTSAQWKELERQAMIYKYMV ASVPVPPHLLLPTADASAPHSPLGSGCGGIFNLEFGNNKDPELGRCKRTD GKKWRCSRDVAPHQKYCERHMHRGRPRSRKPVEGPPNNIKKKTRLHLP THPTPTPKQLLRSTVPPNEIENSSSLLLDSPKTSEHFYVLPYGEQSNRGLDW MVEGEMVNMDTSEQQWQQLMTEGSIYSTTAPSIFQQSYGEEEPMNLLSL PDIVGNSPNEYNLFLHSYQPPRDFIDAWSNDSSNNENNNNHSSVTAANG NLSPSSLNFSMAMAVGNSLDHEIGQNQMGLDVARAINYHQKSQVSSWL HPVCTPGGPLAEVLRPSSVAIGSNPGSPCRRN
>HhGRF3	MDLGGNVVGLISSFSDTTTSNVFASDLEAKQKWYGSGFLKQERLVPISTEP DDCIRDFKVVKNTDDFSQSKAMLLHQKQQQQQQKNLSLLRSNSSPLFY DGQQQMLSFSSPNSQPVTLPYYHHSSTPYSRNTGYDSGGLNAANMHGIIT GVRGPFTPSQWMELEHQALIYKYITANSPVPSNLLSPIRKALESAGFSSFPG AHLRPNTLGWGAFHLGFSNNTDPEPGRCRRTDGKKWRCSRDAVADQK YCERHVNRGRHRSRKPVEGQTGHSVSGTTNTTAKFLPMSSSTSAAAVVPA SGTSNSLGLSNHQLNNLQHGAPNPSASAHINRSFLNKANVGENTQDTT GLTMLSPTIGLKENQFSSQKQHNPYQESSRTEFGLVCSDSLLNPFQKSSSLI NCRSYGSSEDLNDRENKSQHSLRQFMDDWPKNQSERSAISWPDIDMQSD RTQLSISIPMAASDFMSSTSSPTNEKLALSPLRLSRELDSTQMGLGMNGIN NETNQRQASWIPISWENSMGGPLGEVLHSTNNSAGDCNNTTGLNLMTE GWDSSPRFASSPTGVLQKTTFRSLSNSSAGSSPRTENNLIGANLISSSLPAL
>HhGRF4	MDFGSDVGLLSPPSNTTTTAFAYSADPEIKQKWYGSGFFKQEKSNSSSLFS DGQQQMLSFSSPNLQPVTLPCYHETASPYTRNTGYGYGGLNAANMHGT VTGIKGPFTPSQWMELEHQALIYKYITANSPVPSNLLNPIRKALESAGFSGF PGSHLRHNTLGWSPFHLGFSNNTDPEPGRCRRTDGKKWRCSRDAVADQ KYCERHVNRGRHRSRKPVECQTAHSISGTTNTTAKFLPLSTSTSAAAVVPD SGTSNSLGLSDHQLNNLQHGAPNPSASPHLKRSYMNKVNVGEKIQEQQ NLYHEPSRTEFGLVCSDSLLNPFQKSSSLINCRSYGSNDDLNDRQNKSQHS LLQFMDDWPKNQSERSAMTWPDIDMQSDRTQLSISIPMAASDFMSSTSSP TNEKLALSPLRLSRELDSSQMGLGMNTIINETNQRQANWIPISWENTMG

	GPLGEVLHSTNSSAGDCKNSSALNLMTEGWDSSPRLASSPTGVLQKTTFR
	SFSSSSLGSSPRTENNLLCTNLIGASLPAL
>HhGRF5	LFHEENCLVSILMGMKNSPSIDSDIIVGFGLKLRSTESFPKKKKTSYYNDHH
	RPFSPLQSMDANIAGGMATTLGYPGGFTSAQWKELERQAMIYKYMVASV
	PVPPALLLPFTHTADSSASHPPYPEPGRCKRTDGKKWRCSRDVAPHQKYC
	ERHMHRGRPRSRKPVEVPPNTTTKKTRLHLPTAPTSQAPTNPSPRSSIPPN
	EITNPSSLVPNNNTKDNEDISAPHRDQQPNRGLDWMMEGEMVTMDTSE
	QQWQQLMSEGSIYNTSTSSIFQQDYGDEPMNLLSLPEICNSHNDEYNLFL
	NPYQPPRGFIDAWSNDNNPNSENNNNESSVAANANLSPSSLNLSMAMG
	VGNSLDEEMGQIQMGLGVSSWLSPVPWMGSTPGGPLAEVLRPSSLAIGS
	NPGSPYAGKNFDALSPPATSASSPSGVLQRTVLSLSDSSVCNSPTLAATSTA
	APEVLGNSGNS
>HhGRF6	MDFHLKQWREPQHESDPDQQSSAAAKLPRQLLLDSYHQQQQSSEASAV
	LPLFTPEPTTTNPSADSTTTRSSRMGGSCYFSMAQWQELELQALIFRHMLA
	GAAVPPELLHLLKKSLSLTSPSPYYMPHHLQHYSHYQPALLQSGYWGRG
	AMDPEPGRCRRTDGKKWRCSRDVVSGHKYCERHMHRGRNRSRKPVEIP
	TPASADANNNSNGVGVLMKTNTNLPPIAAQPLTAAMGVGGTNFGLSGP
	SHSFDLLHFNQRPSSESITETKGLFRSHNNELSEDDKSGGQILRHFFDDWP
	RSVQEPENTMNNASPGTSLSISVPGNPSSDFSLKLSTGGNRDEPGPAERHV
	ERERAHLNWGAPTGWGTNQMGGPLAEALRSSTSTSSPTSVLHQLRAEAS
	FIST
>HhGRF7	MHGSFTRCKGPFTPSQWLELELQALIYKYILANVPIPSHLLIQIRKSLNPFV
	YVGSSSGSYAPNSIGWGTFHLGLSGSTDPEPGRCRRTDGKKWRCSREAVP
	DQKYCERHINRGRHRSRKPVESHNGQVVTGTTNTKVVPIASSPSASVMC
	NSGWSKNLGQTQHQFKSLQPDTANPSINASVNRMQGTQGQSMISPIINL
	MSKDDPFSIPTQLVPIGGYSRPEFSVVSSASLIYPSDKSSYSNPRNCDSFLSFN
	DQETHHEQPLRHFMNDLPKEQSDRLTVSWPEEFKSDWAQLSMSIPMASC
	DFSSCSNSPRQEKFTLTPLSLPCELDSINLSLGALNDPIKKPTNWIPVTWGN
	SMGGPLGEALNSINSSVEANKSSLVLNLMTEAWDGRPQLGSSPTGVLQK
	AAFVSVSNSSSGSSPGADYKASGSISDDALAGSVSIPL
>HhGRF8	MMSTSNARNNRFPFTASQWQELEHQALIFKYMVSGMPIPPDLLFTIKRSL
	DSSKLILHQPQHIGWNCFQMGFGRKIDPEPGRCRRTDGKKWRCSKEAYP
	DSKYCERHMHRGRNRSRKPVETTSTNSTSSREISLTTAIPISSITRNNPNPST
	PYNNIQTNSTSHQSLSSITSYNNTPLQNPFLYPHSSSSRPSGIGLESSPYSQTD
	KDYRNSSYGHGMKEEIGEHVFFSPSSGTMRSMSGSSVGDSSWHLEPLKMD
	GSPLKQRTCSGLQSGYSYLQLNSKEKRDQHYYVMDDHVQLKMERGDEP
	QKVMHHFFDEWPKNKDSWLETEDKLPNHGQVSATQLSISMPRSSHHDF
	FMTHNEK
>HhGRF9	MSGSTSSLVAAGDGFRPPFTAVQWQELEQQALIYKYLMAGLPVPPDLVATI
	RRSLEALSARFFHHPTVGYCSYYGKKFDPEPGRCRRTDGKKWRCSKDAYP
	DSKYCERHMHRGRNRSRKPVESQSTSQSLSTAMSHIATGSSSGGGSFQGSG
	SGSFQNLPLYSVVNSESLCYGSNVSKLQMDQSSPYGINNKEYRYLQGLTPD
	ADEHNFSLEASASARDLGMDTNTDSKWRLMPSQVRSTSLLRPRNDSHLQ
	GNSTQMHLPQAYELDAAMSKERQQHCFLGNDIGSPGPVKQEQYLMRPF

	FDEWPKTRGSWSDLDDDRCNKNTFSTTHLSISVPMASSEYSARSAHSPDG ELQVPVHNVCP
>HhGRF10	MSGSSSSVAAAGDGFRSPFTAVQWQELEQQALIYKYLMAGLPVPPDLLLQ IRRSLEALSARFFHHPALGYCSYYGKKFDPEPGRCRRTDGKKWRCSKDAY PDSKYCERHMHRGRNRSRKPVESQTTSQSLSTAMSHIPTGSSSGGVSFQSS CSGRFQNQPYSIVNSESLCYGSNASKLQVEPSPYGINNKEYRYLQGLTSDA DEHNFSQEASGSERGLGMDNNIDSTWRLMPSQLHSSSFLKPRNDFHLQR KSTQLHFPQAYELDAAMSKQRQQHYFFGSDIGSSGPVKQEQHSMRPFFD EWPKTRESWSNLDGNKNAFCTTQLSISTPVAPSEYSSRSACSPDDA
>HhGRF11	MMSTTTARNNTFPFTASQWQELEHQALIFKYMLSGMPIPPDLLFTIKRSLD SSLSSNLILHQPQHIGWNCFQMGFGRKIDPEPGRCRRTDGKKWRCSKEAY PDSKYCERHMHRGRNRSRKPVETSSTNISTKDISLPTAIPISSITRNNPPNPN PTTSPYNVQTNSTSHQSLTSIASYNSTQLNNPFLYPHSSSSRPSGIGLESGPY SHTDKDYRNGSYDHGMKEEIDEHVFFSESSGTMRSISGSSVGDSSWHLEPL TMGGSTLKQRTCSGLQNGYSYLQLTSKEKQDQQHYYVLDDDVPLKMDR DNEPQKVMHHFFDEWPKNKDSWLETEDKLSNHGQVSTTQLSISMPKSS HHDFFMTQKWYLKF
>HhGRF12	MSSVDGSGLLIPSEQDWKDSKSISLADDPQLVAPTGILLRSTNSLLAAGQP TMLSFSSPNPLSTKNGAAPPYRSIPETPIIFCQQGGYGYGGSNGGMHGSFT RCKGPFTSSQWLELELQALIYKYILANVPIPSHLLIQIRKSLNPFLYVGSSWG SYAPSSIGWGAFHLGLSGSTYPEPGRCRRTDGKKWRCSREAVPDQKYCER HINRGRHRSRKPVEGHNGQVVAGTTNTKVVPIASSPSASVMCSSGTSKSL GQTQHQFKSLQPTTANPTTNAVVNGMHGTSCQSTLSPIINLKSKDNLFSIP KQLVPIGGSSQPEFSVVSSAASLVHPSDKSSYINPRNCDSFLSFNDQETHHE QPLRHFMNDLPKNQSDCSTVSWPEEFKSDWTQLSVSIPMASCDFSSCSNS PCQEKFTLAPLSLPCELDSIDMSLGALNDSIKKPTNWIPVTRGNSMGGPLG EALNSTPSSVEANNKSSLVLNLMTEEWDGRLMTEEWDGRPQLGSSPTGV LQKATSVSVSNSSSGSSPRADYKASGSLSDDVPCLASTSSVAIPL
>HhGRF13	MSGSSSSVAAAGDGFRPPFTAVQWQELEQQALIYKYLMAGLPVPPDLVVS IRRSLEALSARFFHHPALGYCSYYGKKFDPEPGRCRRTDGKKWRCSKDAY TDSKYCERHMHRGRNRSRKPVESKSTSQSLSTAMSHIATGSNSGGGTFQSS GSGSFQNLPYTVVNSESLCYGSNVSKLQVEPSPYGINNKEYRYLQGLITDA DEHNFSPETSGSSRGLGMDNNIDSTWRLMPSQLHSSSSLKPRNDSHFQGK ATQLHFPQAYELDAGMSKQRQQHCFFGNDIGSSGPMKQEQHLMRPFFN EWPKTRESWSDLDNNKNTFSTTQLSISTPVAPSEYSSRSACSPDDA
>HhGRF14	MMSTTARNNKLIFTASQWQELEHQALIFKHMVSGMPIPPDLLFTIKRSLDS SLSSKLILHQPQHIGWNCFQMGFGRKIDPEPGRCRRTDGKKWRCSKEAYP DSKYCERHMHRGRNRSRKHVETSSTNISTRDISLRTAIPISSITRNNPPNPN HTTSPYNVQTNSTSHQSLTSIASYNSTPLNNPFLYPHSSSSCPSGIGLESGPY SHTDKDYRYGHGMKEEIDEHAFFSESSGTMRSISGSSVGDSSWHLEPLTM GGSTLKQRTYSGLQNGYSYLQLTSKDKQDQQHYYVLDDDVPLKMDRDD EPQKVMHHFFDEWPKNKDSWLETEDKLSNHGQVSTTQLSISMPKSSHH DFFMTHKWYLNFNLSTAKYQSFSAF
>HhGRF15	MEPQSLLKTSPTDLLGERRKGDKLLARNNNWNKGRIEGEEGGLVKEELQ

	FIELGLGICADGYGGPSQVTIKQGKGTPPITANQLHELNQQALIFKYIVAG<br>TPVPFPLVFPIWRSVASSFGFVNGGIYNQFPSFIGFSPRGIDHRCLMDPESGR<br>CRRTDGKKWRCGKNVVPDQKYCERHMHRGRMRSRKPVEAAENISQSN<br>TPTTTCSNKTKQTIKSHDPTDTNTKFFPVPGNHQFKMPSFKRSNGFPATV<br>ASTATSSAKGKSRGTGNVFVKSVTSKPGAINNINLNNCSTDITTTTVTNTF<br>HNADTDNVISRNNRKYVDGKDADFNYINDKKNNNRNSKNGNNESDFV<br>TPGFVFTLKSVRHDDTSSSKSSFDHRCVAEVEPQRCRRTDGKKWWCSRDV<br>VPQQNYCDSHMHRGAKRLTVSSEAVTVAVASPPVAIPKNDHINLNITVDS<br>PQLTSDDVNDSTSGSSSDATTVTDENTSVSHLLALSP
>HhGRF16	MDFGSDVGLLSPLSNTTTAFASSSDPEAKQKWYGSGFFEQGRSNSSSLFSD<br>GQPQMLSFSSPNLRPVVLPYYHQTASPCARNTVYGSVCLNAANMHGMV<br>TGVKGPFTPLQWMELEHQALIYKYITANSPVPSNLLNPIRKALESAGFSSF<br>PGAHLRPNTLGWGAFHLGFSNNTDPELGRCRRTDGKKWRCSRDAVADQ<br>KYCERHVYRGRHRSRKPVEGQTAHSVSRTTRTTAKFLPMSSSTSAASVVP<br>ASSTSNSLGLSHHQLNSWHHGAPNPSASPHLNRSYMNKANVGEKIQEQ<br>QNPYHEPSRTEFGLVCSDSLLNPFQKSSSLINCRSYGSTDDLNDRQNKSQH<br>SLLQFMDGWPKNQSKHEAMSWPDIDMQSDRTQLSISIPMAASDFMSSTSS<br>PTNEKLALSPLRLSRELDSTQMGLGMNAIINETNQRQANWIPISWENTM<br>GGPLGEVLNSTNNSAGDCKNTSALNLMTEGWDSSPRLASSPTGVLQKTT<br>FRSLSNSSAGSSPRTENNLLGTNFISASLPAL
>HhGRF17	IMSYYNDYHRRFSPLQSMGGNDESSGPNCNNKEVTRLCSGCCSNIYRDD<br>DAAAATAKPVVVRGVRQPFDISTTTATTTNTTFKSPGGMATTLGYPGGFT<br>LAQWKELERQAMIYKYMVASVPVPPDLLLPFTHTADSSASHPPLGGGCGI<br>FNLKFGNNKDPEPGRCKRTDGKKWRCSRDVAPHQKYCERHMHRGRPRS<br>RKPVEVPPNTATTKKTRLHLSTAPTSQVPTNPSARSSIPPNEITNPSSLVLN<br>NTKNNEDISMVTMDTSDQQWQQLMSEGSIYNTSNPSIFQQDYGEPMNLL<br>SLPEIGNSSNDEYNLFLNPYQPPRDFIDAWSNDNNPNSENNNNESSVAAI<br>GNLSPSSLNLSMAMAVGNSLDEEMGQIQMGLGGVRDTNHHRKSQVSSW<br>LSPVPWMGSTPGGPLAEVLRPSSLAIGSNPGSPYAGKNCDAISPPATSASSP<br>SGVLQRTMLSLSDSSVCNSPTLAATSTAAPEVLGFRWPS
>HhGRF18	MDFHLKQWREQQHESDPEQQSSAAAKFPRQLLLDSYHQQQQSSEASAV<br>LPLFTPEPTTKLTITNLSADSTTTSSSRMGGSCFFSMAQWQELELQALIFRH<br>LLAGAAVPPELLHLLKKSLILTSPSSPYYMAHPLQHYSHYQPALLQSGCW<br>GRGAMDPEPGRCRRTDGKKWRCSRDVVGGHKYCERHMHRGRNRSRKP<br>VEIPTPTPTASTSTAGANNNSNGVGVLMKANTNPPPIAAQPLTAIGGGGT<br>NFGLSGPPHSFDLLHFNQSRPSESLTETKGLFRTHNNELSDDDKSGGRILR<br>HFFDDWPRSVQEPENTINNASPGTSLSISVPGNPSSDFSLKLSTGGNRDEP<br>GPEERHVEREQAHLNWGAPSGWGTNQMGGPLAEALRSSTSTSSPTSVLH<br>QLRGDASFIS
>HhGRF19	MMSTTNSKSNRFPFTASQWQELEHQALIFKYMVSGMPIPPDLLFTIKRSLD<br>SSKLIVHPPQHVGWNCFQMGFGRKVDPEPGRCRRTDGKKWRCSKEAYQ<br>DSKYCERHMHRGRNRSRKPVETTSTNNASSREISLPTAIPISSITRSNPNPST<br>PCNNIQTNSTSHQSLSSITSYNNTPLQNPFLYPHSSSRPSGIGLQSSPYSQT<br>DKDYRNSNYDHGMKEEIGEHAFFSQASGTMRSMSGSSVGDSSWHLEPLK

	MGGPPLKQRTCSGLRSGYSYLQLNSKEKQDQHYYVMDDDVPLKTVRDD
	EPQKVMHHFFDEWPKNKDSWLETEDKLPNHGQVSATQLSISMPRSSHH
	DFFMTHNGNLD
>HhGRF20	MKLGELSGNTSSSSAAGDGFRPPFTAVQWQELEQQALIYKYLMAGLPVPP
	DLVVPIRRSLEALSARFFHHPALGYCSYYGKKFDPEPGRCRRTDGKKWRC
	SKDAYPDSKYCERHMHRGRNRSRKPVESQSTSQSSSTAMSHIATGSSSGGG
	IFQSSGSGSFQNLPLYSVVNSGSLCYESNVSKLQMEQPSPYGINNKEYRYL
	QGLTPDADEHNITLEASGSARGLGMDTNTDSTWRLMPSQVHSTSLLKSR
	NDSHLQGNSTQLHLPQAYELDVAMSKQRQQHCFFGNDIGSPSPVKQEQ
	HLMRPLFDEWPKTRGSWSDLDDDRCNKNTVSTTQLSISIPMAPSEYSARS
	AHSPDDD
>AtGRF1	MDLGVRVSGHETVSSPGQTELGSGFSNKQERSGFDGEDCWRSSKLSRTST
	DGFSSSPASAKTLSFHQGIPLLRSTTINDPRKGQEHMLSFSSASGKSDVSPY
	LQYCRNSGYGLGGMMNTSNMHGNLLTGVKGPFSLTQWAELEQQALIYK
	YITANVPVPSSLLLSLKKSFFPYGSLPPNSFGWGSFHLGFSGGNMDPEPGR
	CRRTDGKKWRCSRDAVPDQKYCERHINRGRHRSRKPVEGQNGHNTNA
	AAAASAAAASTAAAVSKAAAGTSAVAMRGSDNNNSLAAAVGTQHHTN
	NQSTDSLANRVQNSRGASVFPATMNLQSKETHPKQSNNPFEFGLISSDSL
	LNPSHKQASYATSSKGFGSYLDFGNQAKHAGNHNNVDSWPEELKSDWT
	QLSMSIPMAPSSPVQDKLALSPLRLSREFDPAIHMGLGVNTEFLDPGKKT
	NNWIPISWGNNNSMGGPLGEVLNSTTNSPKFGSSPTGVLQKSTFGSLSNS
	SSASSTIIGDNNNKNGDGKDPLGPTTLMNTSATAPSL
>AtGRF2	MDIGVHVLGSVTSNENESLGLKELIGTKQDRSGFIGEDCLQRSLKLARTTT
	RAEEEENLSSSVAAAYCKTMSFHQGIPLMRSASPLSSDSRRQEQMLSFSDK
	PDALDFSKYVGLDNSSNNKNSLSPFLHQIPPPSYFRSSGGYGSGGMMMN
	MSMQGNFTGVKGPFTLTQWAELEQQALIYKYITANVPVPSSLLISIKKSFYP
	YGSLPPSSFGWGTFHLGFAGGNMDPEPGRCRRTDGKKWRCSRDAVPDQ
	KYCERHINRGRHRSRKPVEVQSGQNQTAAAASKAVTTPQQPVVAGNTN
	RSNARASSNRSLAIGSQYINPSTESLPNNRGVSIYPSTVNLQPKESPVIHQK
	HRNNNNPFEFGHISSDSLLNPNTAKTYGSSFLDFSSNQEKHSGNHNHNS
	WPEELTSDWTQLSMSIPIASSSPSSTHNNNNAQEKTTLSPLRLSRELDLSIQ
	TDETTIEPTVKKVNTWIPISWGNSLGGPLGEVLNSTTNSPTFGSSPTGVLQ
	KSTFCSLSNNSSVSSPIAENNRHNGDYFHYTT
>AtGRF3	MDLQLKQWRSQQQQQHQTESEEQPSAAKIPKHVFDQIHSHTATSTALPL
	FTPEPTSSKLSSLSPDSSSRFPKMGSFFSWAQWQELELQALIYRYMLAGAAV
	PQELLLPIKKSLLHLSPSYFLHHPLQHLPHYQPAWYLGRAAMDPEPGRCR
	RTDGKKWRCSRDVFAGHKYCERHMHRGRNRSRKPVETPTTVNATATSM
	ASSVAAAATTTTATTTSTFAFGGGGGSEEVVGQGGSFFFSGSSNSSSELLHL
	SQSCSEMKQESNNMNNKRPYESHIGFSNNRSDGGHILRPFFDDWPRSSLQ
	EADNSSSPMSSATCLSISMPGNSSSDVSLKLSTGNEEGARSNNNGRDQQN
	MSWWSGGGSNHHHHNMGGPLAEALRSSSSSSPTSVLHQLGVSTQAFH
>AtGRF4	MDLQLKQWRSQQQNESEEQGSAATKISNFFFDQIQSQTATSAAAAPLPLF
	VPEPTSSSSFSCFSPDSSNSSSSSRFLKMGNFFSWAQWQELELQALIYRYML
	AGASVPQELLLPIKKSLLHQSPMHFLHHPLQHSFPHHQPSWYWGRGAM

	DPEPGRCKRTDGKKWRCSRDVVAGHKYCDRHIHRGRNRSRKPVETATTT ITTTATTTASSFVLGEELGHGPNNNHFFSSGSSQPLHLSHQQSCSSEMKQE SNNNKRPYEANSGFSNGRSDDGHILRHFFDDWPRSSDSTSSPMSSSTCHLS ISMPGNNTSSDVSLKLSTGNEEEEENMRNNNNNEREQMNWWSNGGNHH NNMGGPLAEALRSASSTSSVLHQMGISTQVFH
>AtGRF5	MMSLSGSSGRTIGRPPFTPTQWEELEHQALIYKYMVSGVPVPPELIFSIRRS LDTSLVSRLLPHQSLGWGCYQMGFGRKPDPEPGRCRRTDGKKWRCSREA YPDSKYCEKHMHRGRNRARKSLDQNQTTTTPLTSPSLSFTNNNNPSPTLS SSSSSNSSSTTYSASSSSMDAYSNSNRFGLGGSSSNTRGYFNSHSLDYPYPST SPKQQQQTLHHASALSLHQNTNSTSQFNVLASATDHKDFRYFQGIGERV GGVGERTFFPEASRSFQDSPYHHHQQPLATVMNDPYHHCSTDHNKIDH HHTYSSSSSSQHLHHDHDHRQQQCFVLGADMFNKPTRSVLANSSRQDQ NQEEDEKDSSESSKKSLHHFFGEDWAQNKNSSDSWLDLSSHSRLDTGS
>AtGRF6	MDTLSIKTYLLLSYTFNFPIQIPIFNLSFFFISLSLSLFMATRIPFTESQWEELE NQALVFKYLAANMPVPPHLLFLIKRPFLFSSSSSSSSSSFFSPTLSPHFGWN VYEMGMGRKIDAEPGRCRRTDGKKWRCSKEAYPDSKYCERHMHRGKN RSSSRKPPPTQFTPNLFLDSSSRRRRSGYMDDFFSIEPSGSIKSCSGSAMEDN DDGSCRGINNEEKQPDRHCFILGTDLRTRERPLMLEEKLKQRDHDNEEE QGSKRFYRFLDEWPSSKSSVSTSLFI
>AtGRF7	MDFLKVSDKTTIPYRSDSLFSLNQQQYKESSFGFRDMEIHPHPTPYAGNGL LGCYYYYPFTNAQLKELERQAMIYKYMIASIPVPFDLLVSSPSSASPCNNK NIAGDLEPGRCRRTDGKKWRCAKEVVSNHKYCEKHLHRGRPRSRKHVE PPYSRPNNNGGSVKNRDLKKLPQKLSSSSIKDKTLEPMEVSSSISNYRDSR GSEKFTVLATTEQENKYLNFIDVWSDGVRSSEKQSTTSTPVSSSNGNLSLYS LDLSMGGNNLMGQDEMGLIQMGLGVIGSGSEDHHGYGPYGVTSSLEEM SSWLAPMSTTPGGPLAEILRPSTNLAISGDIESYSLMETPTPSSSPSRVMKK MTSSVSDESSQV
>AtGRF8	MRMLLGIPYVDKSVLSNSVLERGKQDKSKLLLVDKCHYELDVEERKEDFV GGFGFGVVENSHKDVMVLPHHHYYPSYSSPSSSSLCYCSAGVSDPMFSVS SNQAYTSSHSGMFTPAGSGSAAVTVADPFFSLSSSGEMRRSMNEDAGAAF SEAQWHELERQRNIYKYMMASVPVPPELLTPFPKNHQSNTNPDVDTYRS GMFSIYADYKNLPLSMWMTVTVAVATGGSLQLGIASSASNNTADLEPWR CKRTDGKKWRCSRNVIPDQKYCERHTHKSRPRSRKHVESSHQSSHHNDI RTAKNDTSQLVRTYPQFYGQPISQIPVLSTLPSASSPYDHHRGLRWFTKED DAIGTLNPETQEAVQLKVGSSRELKRGFDYDLNFRQKEPIVDQSFGALQG LLSLNQTPQHNQETRQFVVEGKQDEAMGSSLTLSMAGGGMEETEGTNQ HQWVSHEGPSWLYSTTPGGPLAEALCLGVSNNPSSSTTTSSCSRSSS
>AtGRF9	MKMQSPKMEQEEVEEERMRNKWPWMKAAQLMEFRMQALVYRYIEAG LRVPHHLVVPIWNSLALSSSSNYNYHSSSLLSNKGVTHIDTLETEPTRCRRT DGKKWRCSNTVLLFEKYCERHMHRGRKRSRKLVESSSEVASSSTKYDNTY GLDRYNESQSHLHGTISGSSNAQVVTIASLPSARSCENVIRPSLVISEFTNKS VSHGRKNMEMSYDDFINEKEASMCVGVVPLQGDESKPSVQKFFPEVSDK CLEAAKFSSNRKNDIIARSREWKNMNVNGGLFHGIHFSPDTVLQERGCF RLQGVETDNEPGRCRRTDGKKWRCSKDVLSGQKYCDKHMHRGMKKK

	HPVDTTNSHENAGFSPLTVETAVRSVVPCKDGDDQKHSVSVMGITLPRVSDEKSTSSCSTDTTITDTALRGEDDDEEYLSLFSPGV
>OsGRF1	MDEEKEADSPQPPSKLPRLSGADPNAGVVTMAAPPPPVGLGLGLGLGGDSRGERDVEASAAAAHKATALTFMQQQELEHQVLIYRYFAAGAPVPVHLVLPIWKSVASSSFGPHRFPSLAVMGLGNLCFDYRSSMEPDPGRCRRTDGKKWRCSRDVVPGHKYCERHVHRGRGRSRKPVEASAAATPANNGGGGGIVFSPTSVLLAHGTARAT
>OsGRF2	MPPCLRRWPTTARPRQPRPPPSSPSAAPPRSPRKQREPAATTHFLGSSGACDNTVRRCVWVGGCRGGGGVAMGEDAPMTARWPPAAAARLPPFTAAQYEELEQQALIYKYLVAGVPVPPDLVLPIRRGLDSLAARFYNHPALGYGPYFGKKLDPEPGRCRRTDGKKWRCSKEAAPDSKYCERHMHRGRNRSRKPVETQLVAQSQPPSSVVGSAAAPLAAASNGSSFQNHSLYPAIAGSNGGGGGRNMPSSFGSALGSQLHMDNAAPYAAVGGGTGKDLRYTAYGTRSLADEQSQLITEAINTSIENPWRLLPSQNSPFPLSSYSQLGALSDLGQNTPSSLSKVQRQPLSFFGNDYAAVDSVKQENQTLRPFFDEWPKGRDSWSDLADENANLSSFSGTQLSISIPMASSDFSAASSRSTNGD
>OsGRF3	MMMMSGRPSGGAGGGRYPFTASQWQELEHQALIYKYMASGTPIPSDLILPLRRSFLLDSALATSPSLAFPPQPSLGWGCFGMGFGRKAEDPEPGRCRRTDGKKWRCSKEAYPDSKYCEKHMHRGKNRSRKPVEMSLATPPPPSSSATSAASNTSAGVAPTTTTTSSPAPSYSRPAPHDAAPYQALYGGPYAAATARTPAAAAYHAQVSPFHLQLDTTHPHPPPSYYSMDHKEYAYGHATKEVHGEHAFFSDGTEREHHHAAAGHGQWQFKQLGMEPKQSTTPLFPGAGYGHTAASPYAIDLSKEDDDEKERRQQQQQQQQHCFLLGADLRLEKPAGHDHAAAAQKPLRHFFDEWPHEKNSKGSWMGLEGETQLSMSIPMAANDLPITTTSRYHNDD
>OsGRF4	MFADFSAAAMELGEVLGLQGLTVPSTKEGDLSLIKRAAAGSFTQAAAASYPSPFLDEQKMLRFAKAAHTLPSGLDFGRENEQRFLLSRTKRPFTPSQWMELEHQALIYKYLNAKAPIPSSLLISISKSFRSSANRMSWRPLYQGFPNADSDPEPGRCRRTDGKKWRCSKEAMADHKYCERHINRNRHRSRKPVENQSRKTVKETPCAGSLPSSVGQGSFKKAKVNEMKPRSISYWTDSLNRTMANKEKGNKAAEENNGPLLNLTNQQPTLSLFSQLKQQNKPEKFNTAGDSESISSNTMLKPWESSNQQNNKSIPFTKMHDRGCLQSVLQNFSLPKDEKMEFQKSKDSNVMTVPSTFYSSPEDPRVSCHAPNMAQMQEDSISSSWEMPQGGPLGEILTNSKNPDDSIMKPEARPYGWLLNLEDHAM
>OsGRF5	MQGAMARVRGPFTPSQWIELEHQALIYKYLAANSPVPHSLLIPIRRSLTSPYSPAYFGSSTLGWGSFQLGYSGSADPEPGRCRRTDGKKWRCSRDAVADQKYCERHMNRGRHRSRKHVEGQPGHAAKAMPAAVAAAAASATQPSAPAAHSGGAVAGLAINHQHQQMKNYAANTANPCSLQYSRDLANKHNESEQVQDSDSLSMLTSISTRNTGSLFPFSKQHNPFEVSNSRPDFGLVSPDSLMSSPHSSLENVNLLTSQSLNEQQSSVSLQHFVDWPRTPAQGALAWPDAEDMQAQRSQLSISAPMASSDLSSASTSPIHEKLMLSPLKLSREYSPIGLGFAANRDEVNQGEANWMPMFRDSLMGGPLGEVLTKNNNMEARNCLSESLNLLNDGWDSSSGFDSSPVGVLQKTTFGSVSSSTGSSPRLENHSVYDGNSNLRDDLGSVVVNHPSIRLV

>OsGRF6	MLAEGRQVYLPPPPPSKLPRLSGTDPTDGVVTMAAPSPLVLGLGLGLGGS GSDSSGSDAEASAATVREARPPSALTFMQRQELEQQVLIYRYFAAGAPVP VHLVLPIWKSIAAASSFGPQSFPSLTGLGSLCFDYRSSMEPEPGRCRRTDGK KWRCSRDVVPGHKYCERHVHRGRGRSRKPMEASAAVAPTYLPVRPALH TVATLATSAPSLSHLGFSSASKVLLAHTTTGTTRAT
>OsGRF7	MAMPFASLSPAADHRPSFIFPFCRSSPLSAVGEEAQQHMMGARWAAAVA RPPPFTAAQYEELEQQALIYKYLVAGVPVPADLLLPIRRGLDSLASRFYHH PVLGYGSYFGKKLDPEPGRCRRTDGKKWRCSKEAAPDSKYCERHMHRG RNRSRKPVEAQLVAPHSQPPATAPAAAVTSTAFQNHSLYPAIANGGGAN GGGGGGGGGSAPGSFALGSNTQLHMDNAASYSTVAAGAGNKDFRYS AYGVRPLADEHSPLITGAMDTSIDNSWCLLPSQTSTFSVSSYPMLGNLSEL DQNTICSLPKVEREPLSFFGSDYVTVDSGKQENQTLRPFFDEWPKARDSW PDLADDNSLATFSATQLSISIPMATSDFSTTSSRSHNGIYSR
>OsGRF8	MLSSSPSAAAPGIGGYQPQRGAAVFTAAQWAELEQQALIYKYLVAGVPVP GDLLLPIRPHSSAAATYSFANPAAAPFYHHHHHPSLSYYAYYGKKLDPEP WRCRRTDGKKWRCSKEAHPDSKYCERHMHRGRNRSRKPVESKTAAPAP QSQPQLSNVTTATHDTDAPLPSLTVGAKTHGLSLGGAGSSQFHVDAPSYG SKYSLGAKADVGELSFFSGASGNTRGFTIDSPTDSSWHSLPSSVPPYPMSKP RDSGLLPGAYSYSHLEPSQELGQVTIASLSQEQERRSFGGGAGGMLGNVK HENQPLRPFFDEWPGRRDSWSEMDEERSNQTSFSTTQLSISIPMPRCGSPI GPRLP
>OsGRF9	MMAGGGSGRCLFTATQWQELEHQALIYKYMAAGAPVPPDLLLHLRHR AAAAAAADVDTVPSLAFPPHHLGWGCYGAAAAQYGRRVEDPEPGRCR RTDGKKWRCSREAYGESKYCEKHMHRGKNRSRKPVEMPPPAAAAVYRP SALSISPPPHDADAPSYGAGAGAPLQLHLDSFHASTSPPPSYHRYAHTSSA PLFPSSAAGYGGGWSLSKEHCLTLGGAAADLSLDKPADHHHDATSATTE KPLRRFFDEWPRSDDGRTPWDGTQLSISIPTAAAASPDLAIAGAASRYHS NGDHLRTSE
>OsGRF10	MAAEGEAKKDSASNPPGGGGGGGGGEEEEDSSLAVGEAAVGVGEAGGG GGGGEKADREEEEGKEDVEEGGVCKDLVLVEDAVPVEDPEEAAATAALQ EEMKALVESVPVGAGAAFTAMQLQELEQQSRVYQYMAARVPVPTHLVFP IWKSVTGASSEGAQKYPTLMGLATLCLDFGKNPEPEPGRCRRTDGKKWR CWRNAIANEKYCERHMHRGRKRPVQLVVEDDEPDSTSGSKPASGKATEG GKKTDDKSSSSKKLAVAAPAAVEST
>OsGRF11	MLSSCGGHGHGNPRSLQEEHHGRCGEQQGGGGGGGQEQEQDGFLVRE ARASPPSPSSSSFLGSTSSSCSGGGGGGQMLSFSSPNGTAGLGLSSGGSMQG VLARVRGPFTPTQWMELEHQALIYKHIAANVSVPSSLLLPIRRSLHPWGW GSFPPGCADVEPRRCRRTDGKKWRCSRDAVGDQKYCERHINRGRHRSRK HVEGRKATLTIAEPSTVIAAGVSSRGHTVARQKQVKGSAATVSDPFSRQS NRKFLEKQNVVDQLSPMDSFDFSSTQSSPNYDNVALSPLKLHHDHDESYI GHGAGSSSEKGSMMYESRLTVSKETLDDGPLGEVFKRKNCQSASTEILTEK WTENPNLHCPSGILQMATKFNSISSGNTVNSGGTAVENLITDNGYLTARM MNPHIVPTLL
>OsGRF12	MAMATPTTNGSFLLGSGGYPGAQILSFSSSGHSGNGLDCGSSDVARMQG

	VLARVRGPFTPTQWMELEHQALIYKHIVANAPVPAGLLLPIRRSLHPPVFP<br>HFSSGGILGSSSLGWGSFQLGYSGSADSEPGRCRRTDGKKWRCSRDAVVD<br>QKYCERHINRGRHRSRKHVEGQSSHAAKATVPAIAQPPIGASNGKLSGSH<br>GVSNELTKTLATNRMMLDKANLIERSQDYTNQQHNILQNNTKGDNWSE<br>EMSSQADYAVIPAGSLMNTPQSANLNPIPQQQRCKQSLFGKGIQHDDIQL<br>SISIPVDNSDLPTNYNKAQMDHVVGGSSNGGNNTRASWIPGSWEASIGG<br>PLGEFFTNTSSASDDKGKSRHPPSLNLLADGHTTSPQLQSPTGVLQMTSFS<br>SVPSSTVSSPAGSLCNGLLTSGLVNAQTVQTL

Table S6 Primers used for quantitative real-time PCR.

Gene ID	Forward primer (5′→3′)	Reverse primer (5′→3′)
HhGRF1	CAACACCACTGCCAAGTTTC	TTGAGAGGCCAAGGCTATTG
HhGRF2	GTGATACTGTGTGGGTTTAGGT	CTCGTTAGCTCCGTTGTCATTA
HhGRF3	CAACACCACTGCCAAGTTTC	TTGAGAGGCCAAGGCTATTG
HhGRF4	GTTGGTGGGTCGGTTAATTTG	GCAGAATAAGCAAAGGCAGTAG
HhGRF5	CTCAATGGAAGGAGCTTGAGAG	CAGTGTGGGTAAAGGGTAAGAG
HhGRF6	CTCACCAACTAGCGTTCTACAC	AGAGAGAGAGAGACACGACATC
HhGRF7	CCCATCTGCATCGGTAATGT	CCTTGGGTACCTTGCATTCT
HhGRF8	GAAGCATACCCAGATTCCAAGT	GATGTTGTTTCCACAGGCTTTC
HhGRF9	ACACATTCTCCACCACTCATC	GGGAACTTGTAACTCACCATCT
HhGRF10	GCAAAGGAAGTCCACACAAC	CCAATATCGCTGCCAAAGAAG
HhGRF11	ACTCCTCTCTCTCCTCCAATC	CCCATCAGTTCTTCTGCATCT
HhGRF12	AGCAGTCCAAGAGCTGATTAC	GCCAGAAGGCATGCATTTAC
HhGRF13	AGGACCGTTTACAGGTTTGAG	CCACTCATCTCGCCTTACTTAC
HhGRF14	GTCCTTATTCCCACACAGACAA	CCTCATAGTCCCTGAACTCTCT
HhGRF15	GCAAAGGGACTCCACCTATTAC	GCTAGCTACACTCCTCCAAATG
HhGRF16	CAGCCAGCAGTACATCCAATA	GTGGAGACGCAGAAGGATTAG
HhGRF17	CTCAATGGAAGGAGCTTGAGAG	CAGTGTGGGTAAAGGGTAAGAG
HhGRF18	GGAGGACAGATGGGAAGAAATG	GCCGTAGAAGTAGAAGCAGTAG
HhGRF19	CGTGGCTTGAAACAGAGGATAA	GAAGAAGTCATGGTGCGAAGAT
HhGRF20	GGTGCTCTAAAGATGCCTATCC	AGTCGATGAGGACTGAGAAGTA
β-Actin1	CTTGACTATGAGCAAGAGTTGG	ATCTTCATGCTACTGGGAGC
β-Actin2	GATGGGCAGACAATAGAAATTGG	CTTTTACTCTAGCTGCTTGAGG
β-Actin3	GATGGGCAGACAATAGAAATTGG	CTTTTACTCTAGCTGCTTGAGG

Table S7 Primers used for molecular cloning and vector construction.

	Forward primer (5′→3′)	Reverse primer (5′→3′)
HhGRF10	ATGAGTGGCAGTTCGTCGTC	AGCATCATCTGGGGAGCAAG
OE-*HhGRF10*	AGCTCGGTACCCGGGGATCC	CAAGCTTGCATGCCTGCAGG
	ATGAGTGGCAGTTCGTCGTC	AGCATCATCTGGGGAGCAAG
KO-*HhGRF10*	GATTGGCCAAAAACTAGAGAATCATGG	AAACCCATGATTCTCTAGTTTTTGGCC

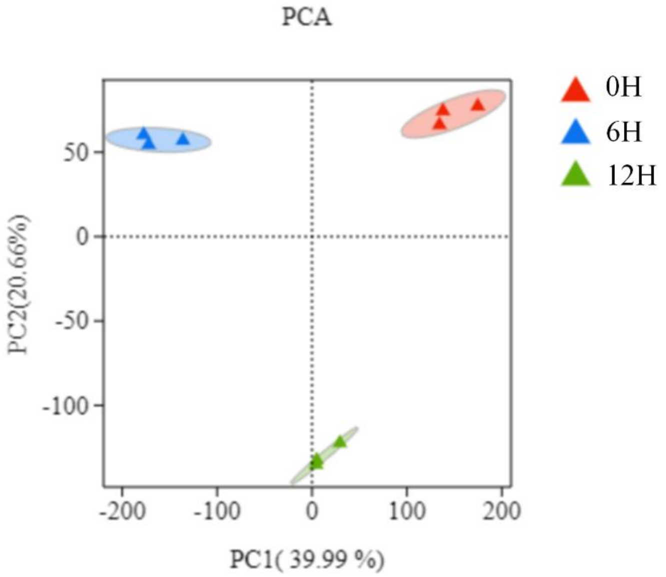

Figure S1 Principal component analysis (PCA) of all samples.

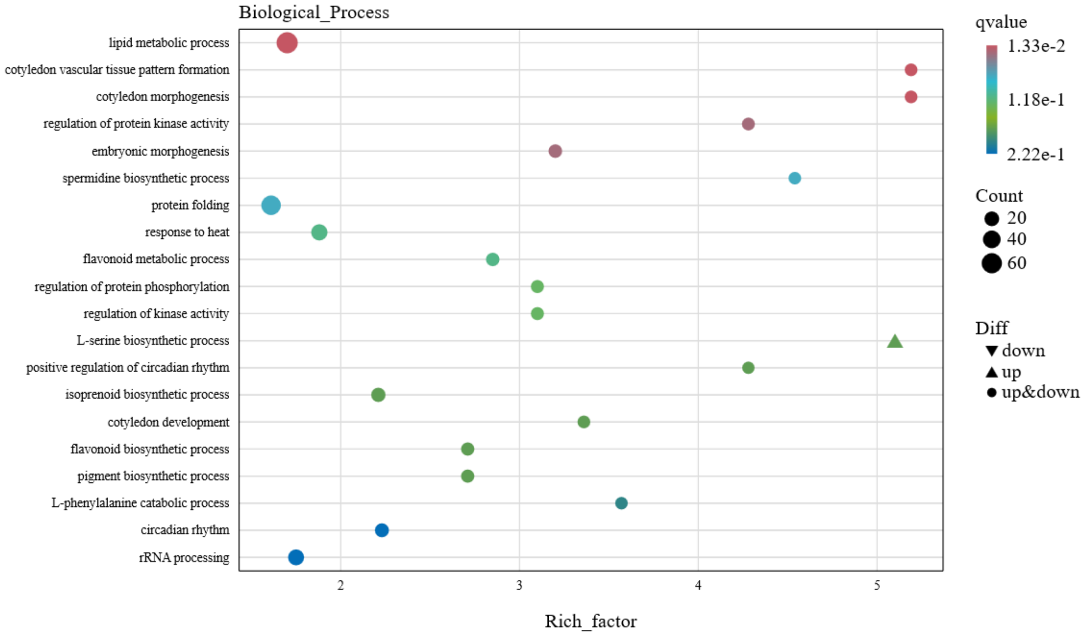

Figure S2 Gene Ontology (GO) enrichment analysis of DEGs.

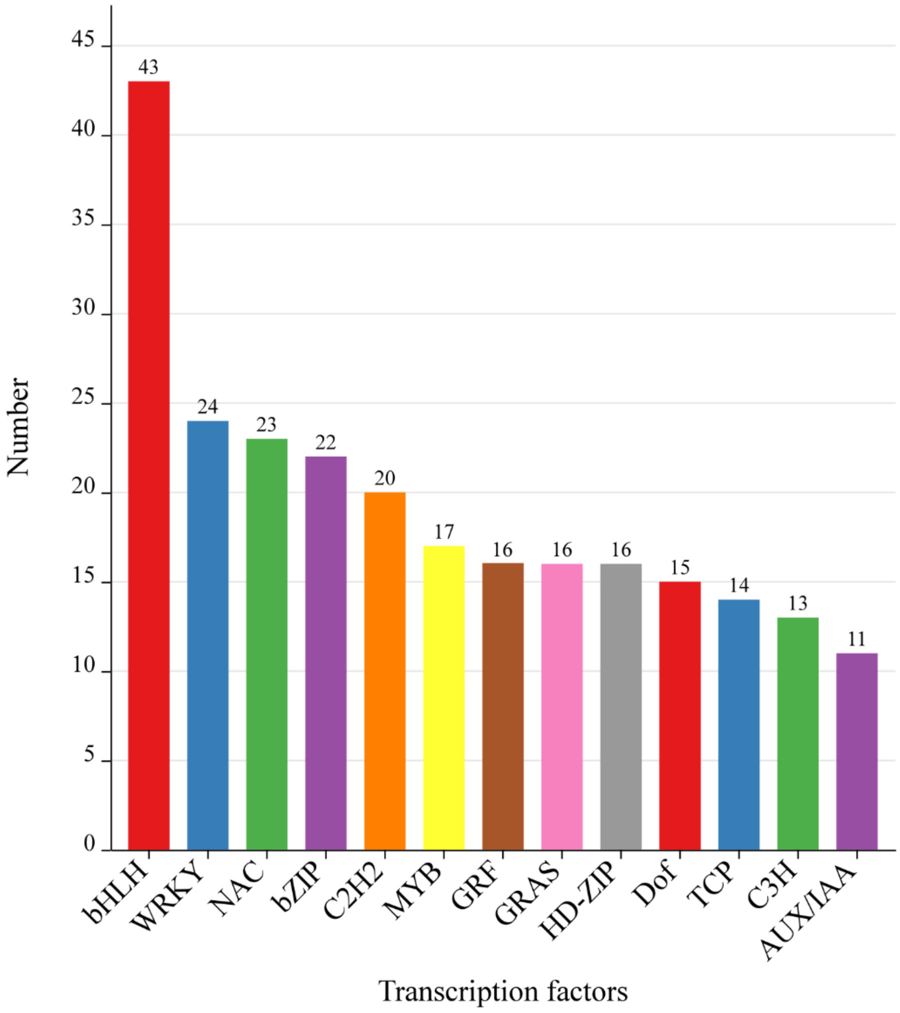

Figure S3 Analysis of differentially expressed transcription factors.

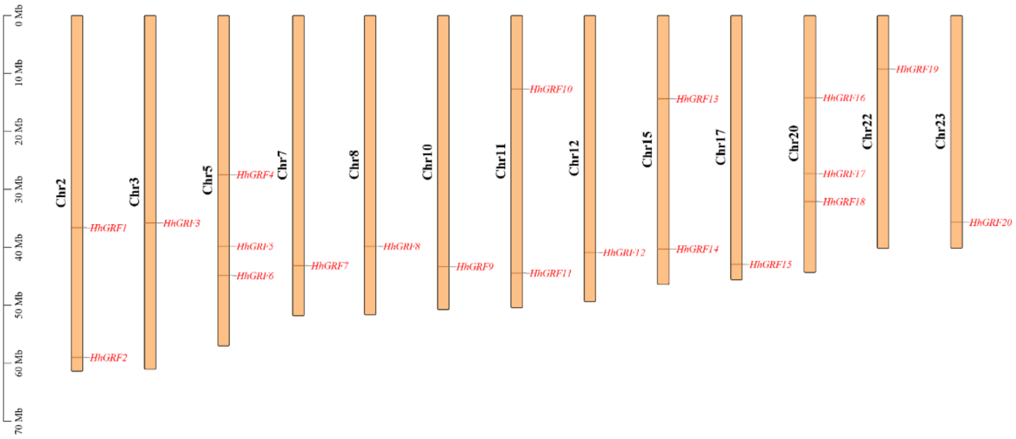

Figure S4 Chromosomal localization of GRF genes in *H. helix*.

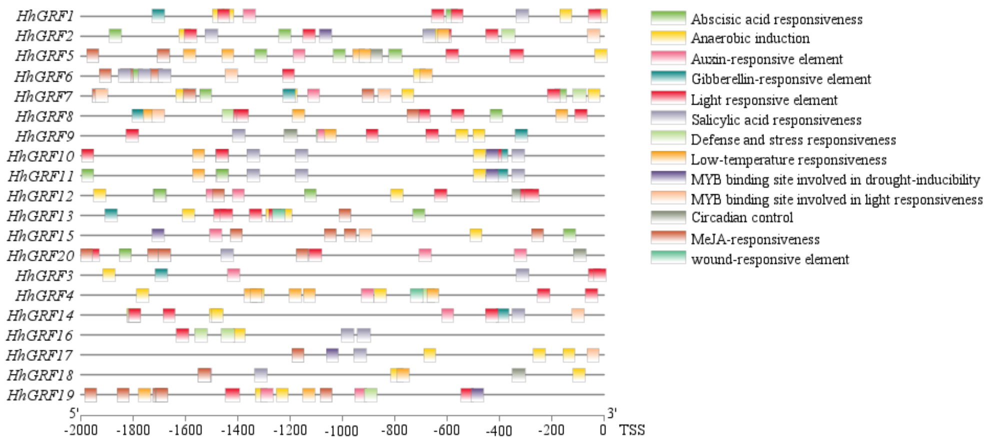

Figure S5 The positions and numbers of cis-regulating element of HhGRF genes in *H. helix*

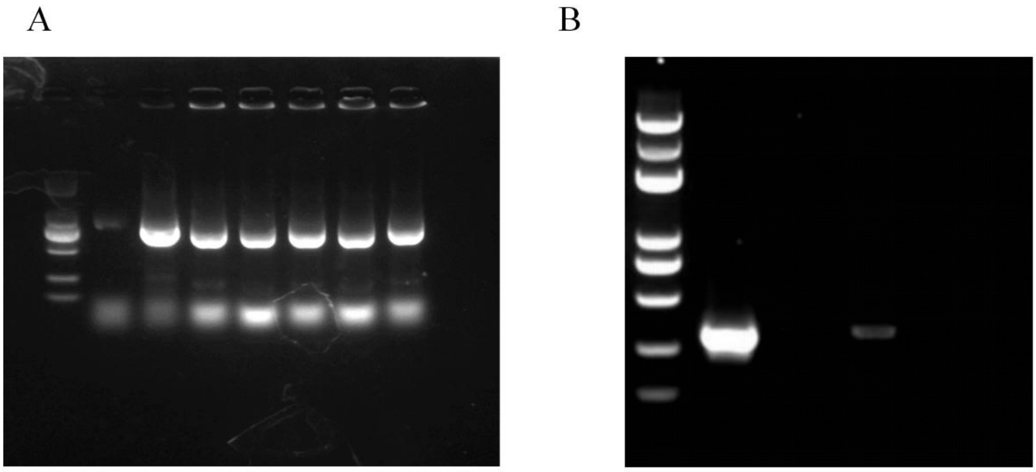

Figure S6 Positive PCR identification of OE (A) and KO (B) transgenic plants.
